# Supplementary material for: Identifying resources used by young people to overcome mental distress in three Latin American cities: a qualitative study
Source: BMJ Open. 2022 Aug 11;12(8):e060340. doi: 10.1136/bmjopen-2021-060340 (PMC9379470; doi:10.1136/bmjopen-2021-060340)
Supplement: Supplementary data [file bmjopen-2021-060340supp001.pdf]

OLA WP1 Arts-based workshops: Topic guide for structured conversations with adolescents and young adults

Objectives: (1) To explore the experiences of emotional and mental health problems, including feelings of depression, anxiety, and stress of young people with emphasis on understanding how these episodes have been recovered and overcome. (2) Explore what role they think arts and creativity had or could have had to protect them and help them recover from anxiety and depression.

a. Breaking the ice

What caught your attention in the theatre?
If theatre is removed from your life, what would you lose?

b. Recognizing mental anguish

We want to know what emotional and mental health problems (anxiety / depression / stress) mean for you.

Have you ever experienced it? If not, have you perceived it in people close to you?

How do you experience it? Can you visualize / express it? Can you communicate it to others? Have you found metaphors or symbols or arts to express these feelings?

Have you found any artistic resource to express these feelings, such as workshops, poetry, music, etc?

c. Coping with mental anguish

Where do you go when you feel distressed? Real places? Fantasy worlds? Are you looking for help in your environment? Do you attend reflection groups? Individual therapy? Group therapy? Do you go to Church? Do you play sport or take physical exercise? Or do you take refuge in yourselves? Do you use your imagination? Do you make art?

How do you physically experience anxiety / distress? What other impact does it have on your life?

Who do they talk to about these feelings? Other young people? Friends? Teachers? Families? Where? When? Who can help best in these moments?

Can you map out the resources (people / places / activities) in your daily life that allow you to reduce distressed state and acquire greater strength in triggering situations? What keeps you safe and comfortable? What significant physical and mental changes do you think you have experienced? How could them describe this mood change?

The recovery process is not always linear or progressive, it involves progress and Setbacks. Can you identify what are the situations or elements that influence those setbacks?

What emotions or feelings do you perceive when moving from anxiety to emotional stability?

d. Understanding the relationship between art and resilience

How do the arts and creative practices build your ability to recover from anxiety and distress as well as to develop resilience to mental pathology?
