# Supplementary material for: Identifying resources used by young people to overcome mental distress in three Latin American cities: a qualitative study
Source: BMJ Open. 2022 Aug 11;12(8):e060340. doi: 10.1136/bmjopen-2021-060340 (PMC9379470; doi:10.1136/bmjopen-2021-060340)
Supplement: Author's manuscript [file bmjopen-2021-060340.draft_revisions.pdf]

BMJ Open is committed to open peer review. As part of this commitment we make the peer review history of every article we publish publicly available.

When an article is published we post the peer reviewers' comments and the authors' responses online. We also post the versions of the paper that were used during peer review. These are the versions that the peer review comments apply to.

The versions of the paper that follow are the versions that were submitted during the peer review process. They are not the versions of record or the final published versions. They should not be cited or distributed as the published version of this manuscript.

BMJ Open is an open access journal and the full, final, typeset and author-corrected version of record of the manuscript is available on our site with no access controls, subscription charges or pay-per-view fees (http://bmjopen.bmj.com).

If you have any questions on BMJ Open's open peer review process please email info.bmjopen@bmj.com

BMJ Open

Identifying resources used by young people to overcome mental distress in three Latin American cities: A qualitative study

Journal:	*BMJ Open*
Manuscript ID	bmjopen-2021-060340
Article Type:	Original research
Date Submitted by the Author:	20-Dec-2021
Complete List of Authors:	Toyama, Mauricio; Universidad Peruana Cayetano Heredia, CRONICAS Center of Excellence in Chronic Diseases<br>Godoy-Casasbuenas, Natalia; Pontificia Universidad Javeriana, Department of Clinical Epidemiology and Biostatistics<br>Olivar, Natividad; Universidad de Buenos Aires, Department of Psychiatry and Mental Health, School of Medicine<br>Brusco, Luis Ignacio; Universidad de Buenos Aires, Department of Psychiatry and Mental Health, School of Medicine<br>Carbonetti, Fernando; Universidad de Buenos Aires, Department of Psychiatry and Mental Health, School of Medicine<br>Diez-Canseco, Francisco; Universidad Peruana Cayetano Heredia, CRONICAS Center of Excellence in Chronic Diseases<br>Gómez-Restrepo, Carlos; Pontificia Universidad Javeriana, Department of Clinical Epidemiology and Biostatistics; Pontificia Universidad Javeriana, Department of Psychiatry and Mental Health<br>Heritage, Paul; Queen Mary University of London School of English and Drama<br>Hidalgo-Padilla, Liliana; Universidad Peruana Cayetano Heredia, CRONICAS Center of Excellence in Chronic Diseases<br>Uribe, Miguel ; Pontificia Universidad Javeriana, Department of Psychiatry and Mental Health<br>Fung, Catherine ; Queen Mary University of London, Unit for Social and Community Psychiatry<br>Priebe, Stefan; Queen Mary University of London, Unit for Social and Community Psychiatry
Keywords:	MENTAL HEALTH, QUALITATIVE RESEARCH, Child & adolescent psychiatry < PSYCHIATRY, Depression & mood disorders < PSYCHIATRY

SCHOLARONE™
Manuscripts

I, the Submitting Author has the right to grant and does grant on behalf of all authors of the Work (as defined in the below author licence), an exclusive licence and/or a non-exclusive licence for contributions from authors who are: i) UK Crown employees; ii) where BMJ has agreed a CC-BY licence shall apply, and/or iii) in accordance with the terms applicable for US Federal Government officers or employees acting as part of their official duties; on a worldwide, perpetual, irrevocable, royalty-free basis to BMJ Publishing Group Ltd ("BMJ") its licensees and where the relevant Journal is co-owned by BMJ to the co-owners of the Journal, to publish the Work in this journal and any other BMJ products and to exploit all rights, as set out in our licence.

The Submitting Author accepts and understands that any supply made under these terms is made by BMJ to the Submitting Author unless you are acting as an employee on behalf of your employer or a postgraduate student of an affiliated institution which is paying any applicable article publishing charge ("APC") for Open Access articles. Where the Submitting Author wishes to make the Work available on an Open Access basis (and intends to pay the relevant APC), the terms of reuse of such Open Access shall be governed by a Creative Commons licence – details of these licences and which Creative Commons licence will apply to this Work are set out in our licence referred to above.

Other than as permitted in any relevant BMJ Author's Self Archiving Policies, I confirm this Work has not been accepted for publication elsewhere, is not being considered for publication elsewhere and does not duplicate material already published. I confirm all authors consent to publication of this Work and authorise the granting of this licence.

For peer review only

Title: Identifying resources used by young people to overcome mental distress in three Latin American cities: A qualitative study

Authors:

Mauricio Toyama[1], Natalia Godoy-Casasbuenas[2], Natividad Olivar[3], Luis Ignacio Brusco[3], Fernando Carbonetti[3], Francisco Diez-Canseco[1], Carlos Gómez-Restrepo[2,4], Paul Heritage[5], Liliana Hidalgo-Padilla[1], José Miguel Uribe-Restrepo[4], Catherine Fung[6], Stefan Priebe[6]

Affiliations:

[1]CRONICAS Center of Excellence in Chronic Diseases, Universidad Peruana Cayetano Heredia, Lima, Peru

[2]Department of Clinical Epidemiology and Biostatistics, Pontificia Universidad Javeriana, Bogotá

[3]Department of Psychiatry and Mental Health, School of Medicine, Universidad de Buenos Aires, Buenos Aires, Argentina

[4]Department of Psychiatry and Mental Health, Pontificia Universidad Javeriana, Bogotá, Colombia

[5]School of English and Drama, Queen Mary University of London, London, UK

[6]Unit for Social and Community Psychiatry (WHO Collaborating Centre for Mental Health Service Development), Queen Mary University of London, United Kingdom

For peer review only - http://bmjopen.bmj.com/site/about/guidelines.xhtml

Abstract:

Introduction: Anxiety and depression are the most common mental health disorders among young people living in large urban settings of Latin America. However, between 50 and 60% of affected adolescents and young adults manage to overcome their distress within the first year. The main objective of the study was to explore which resources and activities help young people living in deprived urban environments in Latin America to recover from depression and/or anxiety.

Methods: A qualitative study was conducted in Bogotá (Colombia), Buenos Aires (Argentina) and Lima (Peru) with adolescents, young adults and professionals with experience working with young people. A total of 18 online focus groups and 12 online structured conversations embedded into arts workshops were conducted. The focus group discussions and structured conversations were recorded, transcribed, coded and analysed using inductive content analysis.

Results: Eight categories captured the resources and activities that were reported by young people as helpful to overcome mental distress: i) Personal resources, ii) Personal development, iii) Spirituality and religion, iv) Social resources, v) Social media, vi) Community resources, vii) Activities (sub-categorized into artistic, leisure, sports and outdoor activities) and viii) Mental health professionals. Personal and social resources as well as artistic activities and sports were the most common resources identified that help adolescents and young adults to overcome depression and anxiety.

Conclusion: Despite the different contexts of the three cities, young people appear to use similar resources to overcome mental distress. Policies to improve the mental health of young people in deprived urban settings should address the need of community spaces where young people can play sports, meet, and engage in groups, and support community organisations that can enable and facilitate a range of social activities.

Strengths and limitations

- To our knowledge, this is the first study to explore resources of young people to overcome mental distress in lar urban environments across three Latin American countries. Using the same language facilitated the analysis across countries and allowed for direct comparisons.
- The study combined different perspectives (adolescents, young adults, professionals) and methods (focus groups, structured conversations embedded in arts workshops) providing a different approach to engage young people and encourage them to express their views.
- The recruitment of a substantial proportion of participants was done through arts organisations which may have biased the findings, leading to a particularly strong emphasis on arts activities.
- The study was conducted during the Covid-19 pandemic which may have influenced the views of young people as to what they find helpful.
- Focus groups and structured conversations were conducted online, which may have

For peer review only

influenced the interaction between the facilitators and the participants and thus the results.

Key questions

What is already known?

- Young people living in deprived urban neighbourhoods are exposed to various risk factors for developing mental disorders; however, around half of those who develop depression and anxiety recover within a year.
- Personal and social resources that help to overcome mental health problems have been studied for different types of adversities and in different groups, but not for young people in deprived urban environments in Latin America.

What are the new findings?

- Young people report a wide range of resources that help them to recover from mental distress, and the resources are largely similar across three different cities (Bogotá, Buenos Aires, Lima).
- The resources comprise personal (e.g. self-awareness, self-esteem, empathy and coping strategies) and social resources (e.g. support from friends and family and belonging to a group with similar interests) and can be grouped into eight categories.
- Sports and arts activities, often in groups, were commonly mentioned as a resource.

What do the new findings imply?

- Policies to improve the mental health of young people in deprived urban neighbourhoods in Latin America should promote personal and interpersonal skills, facilitate support from peers and groups, and ensure space for and access to sport and arts group activities.

Introduction

Adolescence and young adulthood represent a period of growth and development. Young people experience physical changes as well as changes in their cognitions, emotions and social environment (1,2). At the same time, cultural, socioeconomic, and environmental influences, including poverty, low education (3,4), violence (5), or substance use (6), make adolescents and young adults particularly vulnerable to developing mental health problems.

According to the World Health Organization (WHO), it is estimated that between 10% and 20% of young people experience mental disorders, most commonly depression and anxiety disorders (7). Depression represents a common of illness and disability in this group, can be associated with substance abuse, aggressiveness, eating disorders, and is one of the main risk factors for suicide. Anxiety can be accompanied by irritability, dizziness, chest pain, insomnia, fatigue, and social

For peer review only

fears, and is the ninth cause of illness and disability in this population (8). Poor mental health at young age is associated with further health and social problems such as poorer academic achievement, early school drop-out and substance abuse (9).

Young people living in major cities across Latin America can be exposed to various risk factors for developing mental disorders, such as poverty, violence and social inequality (10-13). The percentage of young people with depression and/or anxiety has been reported to range from 17% in Colombia (14) to 26% in Argentina (15). However, longitudinal studies have found that among young people who suffer from depression and anxiety, between 50 and 60% manage to overcome their distress within one year (16,17).

The ability to overcome mental distress – sometimes referred to as resilience - is linked to personal resources such as motivation and hope and to social resources such as support from peers and access to activities in the surrounding environment (18, 19). In children and young adults, arts activities can be an important social resource (20). In Latin America, studies about resources to cope with adversity have been carried out in the context of social violence (21,22), poverty (23), forced displacement (24) and medical conditions (25), but to our knowledge no study has explored the resources that help young people in this region to overcome mental distress.

The aim of this study is to explore which resources and activities help young people in large cities in Latin America to recover from mental distress in form of depression and/or anxiety, using both conventional focus groups and arts workshops.

Methods

Study design

This qualitative study was conducted within the context of a larger research programme called "Building resilience and resources to reduce depression and anxiety in young people from urban neighbourhoods in Latin America (OLA)". The overall aim of the programme is to identify which characteristics, resources and activities help young people living in urban environments in Latin America to prevent or recover from depression and/or anxiety (26). The programme is a collaboration between Queen Mary, University of London (UK), Universidad de Buenos Aires (Argentina), Pontificia Universidad Javeriana in Bogotá (Colombia) and Universidad Peruana Cayetano Heredia in Lima (Peru). The researchers work in close partnerships with the following arts organisations: Crear Vale La Pena (Argentina), Fundación Batuta and Familia Ayara (Colombia) and Teatro La Plaza (Peru). These organisations specialize in building creative environments, using classical music, rap music, street art, dance, theatre and visual arts, respectively.

This study used two forms of group activities for data collection: focus groups including adolescents (15/16 years), young adults (20-24 years) and professionals working with young

For Peer Review Only

people; and arts workshops with young people (15-24 years). The arts workshops were intended as a different approach to engage young people and elicit their views. Towards the end or following the arts activities, all arts workshops included a structured conversation to explore resources to overcome anxiety and/or depression. Because of the restrictions due to the pandemic, all groups were conducted online between August and November of 2020.

A total of 18 focus groups were conducted: six in each country, two for each group of participants (adolescents, young adults and professionals). The focus groups lasted between approximately 90 and 120 minutes. There were also 12 arts workshops: four in Argentina using a combination of creative activities run by the arts organisation 'Crear Vale La Pena'; two in Colombia using rap and graffiti run by 'Familia Ayara' and four with musical activities run by 'Fundación Batuta'; two in Peru using theatre and dramaturgical exercises run by 'Teatro La Plaza'. The length of the workshops varied. They usually lasted between two and three hours. Each focus group and arts workshop had a facilitator and a co-facilitator or technical supporter.

Study setting

The study was conducted in the capital cities of three Latin American countries - Buenos Aires (Argentina), Bogotá (Colombia) and Lima (Peru) – and focused on young people from poorer economic backgrounds.

Participants

Three groups of participants were included in the study: adolescents, young adults and professionals with experience in working with young people.

For the focus groups, the inclusion criteria were: 15-16 years or 20-24 years of age, respectively; willingness to share personal experiences within the group; capacity to provide assent/consent; and, for the adolescents, consent from a parent/legal guardian. Professionals were included if they were at least 18 years of age, had experience of professionally working with young people, were willing to share personal experience within a group, and had capacity to provide consent.

Inclusion criteria for the arts workshops were: 15-24 years of age, current or past participation in activities of the collaborating arts organisations, capacity to provide assent/consent, and, for those under 18 years old, consent from a parent/legal guardian.

Data collection tools

Basic sociodemographic data and previous experience of anxiety and/or depression were collected from all participants in the focus groups on a brief questionnaire, completed by phone or through videoconference (e.g., Zoom).

For peer review only - http://bmjopen.bmj.com/site/about/guidelines.xhtml

Topic guides for both focus groups and structured conversations within the arts workshops addressed the resources used by young people when experiencing distress in form of anxiety and/or depression(See supplementary 1 and 2). The same topic guides were applied in the three countries.

Procedures

Participants were identified through schools, health services, non-governmental organisations, and youth organisations as well as the four participating arts organisations. Potentially interested participants were contacted via phone by the research team to check the inclusion criteria before they were invited to formally participate in the study. The informed consent procedures were conducted through phone or videoconference. Participants were given the options to provide their consent by phone recording, completing the consent form virtually or completing the form on paper and sending a scan or picture to the research team. Adolescents provided their informed assent, and a parent or legal guardian was contacted to receive their permission and consent.

Data analysis

Audio recordings of the focus groups and structured conversations were transcribed verbatim. We conducted an inductive content analysis using the four stages proposed by Bengtsson (27). The research team from each country analysed the transcriptions of their local participants. At least two members of the research teams in each country worked together during the analysis. During the first stage, decontextualization, the research teams familiarised themselves with the data and inductively created codes based on the relevant information they identified. These codes were added to a spreadsheet shared between the teams. During the recontextualization stage, the codes and categories were discussed in weekly meetings to standardize the codes across the three countries to ensure reliability. After the meetings, each team revisited their transcriptions to either re-code their coding to match those identified in other countries, or to code new information they may have missed, based on codes created by the other teams. Furthermore, during the categorisation stage, the codes were discussed and grouped into categories based on similarity of content. These categories were standardized across the research teams. Finally, during the compilation stage, each team conducted a final analysis, summarizing the information extracted for each code in a table. This table was further summarised in a second round to reduce the amount of information. Subsequently, these summaries were combined into a summary encompassing the information from all three countries.

Patient and Public Involvement

Adolescents and young adults (aged 15-24 years old) with knowledge and lived experience of mental distress, including depression and anxiety, were included as part of the Lived Experience Advisory Panel (LEAP) in the three cities where the study was conducted (Buenos Aires, Bogotá, Lima). We received input from the LEAP committee during the design and conduct of the study. The main roles of the LEAP were to provide expert advice to the local research team on all

For peer review only

appropriate aspects of the project, including research materials, methods, results, and dissemination.

Ethical statement

The study protocol and all procedures were approved by the Institutional Review Board (IRB) of Universidad de Buenos Aires on February 10th 2020, Pontificia Universidad Javeriana on March 3rd 2020, and Universidad Peruana Cayetano Heredia on March 31st 2020. Furthermore, it was approved by the IRB of Queen Mary University of London on June 5th 2020.

Results

Participants' characteristics

A total of 185 participants took part in this study: 111 participants (36 adolescents, 35 young adults and 40 professionals) attended the 18 focus groups, on average approximately six participants per group in each country; and 74 young people (29 adolescents and 45 young adults) took part in the 12 arts workshop. The average number of participants in the workshops varied across the three countries with five in Argentina, six in Colombia and nine in Peru.

The socio-demographic characteristics of all groups are shown for each city in Tables 1 and 2.

Table 1. Socio-demographic characteristics of young people in focus groups and arts workshops

**Focus Groups**				
	**Buenos Aires**	**Bogotá**	**Lima**	**Total**
**Age group**				
Adolescents	13 (48%)	9 (50%)	14 (54%)	36 (51%)
Young adults	14 (52%)	9 (50%)	12 (46%)	35 (49%)
**Education level completed**				
Primary school	17 (63%)	9 (50%)	14 (54%)	40 (56%)
Secondary school	4 (15%)	6 (33%)	9 (35%)	19 (27%)
Higher/technical education	6 (22%)	-	3 (12%)	9 (13%)
Other	-	3 (17%)	-	3 (4%)
**Previous experience of anxiety and/or depression**				

For peer review only

Yes	17 (63%)	14 (78%)	18 (69%)	49 (69%)
No	10 (37%)	4 (22%)	8 (31%)	22 (31%)
**Previous experiences with mental health treatment of those who reported previous experience of anxiety and/or depression**				
Yes	9 (53%)	11 (79%)	7 (39%)	27 (55%)
No	8 (47%)	3 (21%)	11 (61%)	22 (45%)
**Arts workshops**				
**Age group**				
Adolescents	9 (45%)	11 (30%)	9 (53%)	29 (39%)
Young adults	11 (55%)	26 (70%)	8 (47%)	45 (61%)

Table 2. Socio-demographic characteristics of professionals participating in focus groups

	**Buenos Aires**	**Bogotá**	**Lima**	**Total**
Psychologists	4 (29%)	4 (29%)	3 (25%)	11 (28%)
Teachers	3 (21%)	6 (43%)	1 (8%)	10 (25%)
Youth workers	3 (21%)	2 (14%)	3 (25%)	8 (20%)
Psychiatrists	2 (14%)	-	-	2 (5%)
Other (e.g., volunteer, researcher, consultant)	2 (14%)	2 (14%)	5 (42%)	7 (23%)

For peer review only

Resources identified

The resources and activities that help young people to recover from mental distress were grouped into 8 categories: I- Personal resources, II- Personal development, III- Spirituality and religion, IV- Social resources, V- Social media, VI- Community resources, VII- Activities (subcategorised into artistic, leisure, sports and outdoor activities), and VIII- Mental health professionals. Due to the large amount of data collected, only the most commonly reported resources are presented here.

I - Personal resources

Young people, adolescents and professionals refer to personal resources as tools to overcome mental health problems. These resources can be allocated to four sub-categories: relationship with oneself, personal traits, coping strategies and passive strategies.

The first sub-category, relationship with oneself, emphasised the importance of self-awareness and self-esteem. Some participants mentioned that having clear goals builds their confidence and helps them overcome problems.

> *"It is very important to value yourself and accept the problem and know how to handle the situation (...) It is also very important to know yourself and know what you like and do not like to know what can help you with the distress" (Female adolescent, Buenos Aires)*

The second subcategory, personal traits, refers to what young people consider to be the key in overcoming mental distress. Most commonly, empathy was mentioned as a helpful trait, but also resilience and motivation.

The third subcategory were coping strategies, including reflecting about problems, accepting and thinking about a difficult situation, controlling emotions and talking to oneself.

The last subcategory 'passive strategies' involved avoiding or isolating themselves from other people when feeling distressed.

> *"When I feel depressed or sad, I make an abrupt change to the situation, I stop what I am doing, I leave and, for instance, listen to music, draw, or, since generally I do not go out, only sometimes, I prefer to be alone, it is how I feel more peaceful" (Male young adult, Bogotá)*

II- Personal development

Personal development comprises activities related to academic, occupational and/or vocational development. Young adults mentioned work, job placement and entrepreneurship as a helpful resource to cope with difficult situations and stay active and motivated. School attendance was perceived by professionals as a protective factor for adolescents.

> *"Young people without access to school, or outside educational activities, without access to a recreational space, are negatively affected. Economic factors influence a lot and a lack of resources is closely related to the economic situation" (Psychologist, Buenos Aires)*

III- Spirituality and religion

Religion and spirituality were reported as important resources by participants from Bogotá and Lima. For spirituality, some participants from Bogotá stated that meditation as a practice helped

For peer review only

them to relax, calm their mind and get rid of negative energy. Some participants from Lima, mostly young women, mentioned exercises related to relaxation, such as breathing exercises, yoga, meditation, and mindfulness.

Religion was suggested as a resource by participants from both cities. In Bogotá, religion was seen as a resource in moments of sadness and depression and a way to seek guidance for solving problems. In Lima, some participants talked about praying or reading the bible and talking to God was regarded in a similar manner as meditating.

> *"I am Christian, but it's not like I go to confession with a priest (laughs). It's more like simply talking with God, I tell Him 'Father, this happened to me'. It's curious because I did not understand very well from a scientific point of view why that happened, but there is research that says if you are talking with someone, even if they don't exist, it is like a direct meditation. That's how I see it." (female, adolescent, Lima)*

Some participants from Bogotá pointed out that religion can make people feel judged or not good enough. Some participants from Bogotá and Lima remarked that young people are not as religious as older people.

<u>IV- Social resources</u>

Social resources focused on receiving support from friends, family and other people, as well as participating in group activities. Participants from all three cities agreed that support from friends is an important source of emotional support for young people. Talking about their problems made them feel better and engaging in activities with friends was a welcome distraction. Professionals highlighted friends and social groups as an important resource, in some cases more important than the family.

> *"I believe it is fundamental, the support, the emotional support that family or friends can provide. Like someone mentioned, a lot of the time you confide in your friends more than in your family" (Male young adult, Bogotá)*

Repeatedly, young people referred to the family as an important source of support, providing helpful advice and making them feel calmer and more relaxed. Family support was seen as a valuable resource to navigate situations of emotional distress. Other social sources of support were partners and teachers.

> *"In my case, I talk more with my family, my mom, my sisters, my dad, mostly about things that happen at work, I always talk to them. First, because they are closer to me and because I believe they have the experience to give me advice, with other issues, maybe outside of work, I may talk to friends, but almost always I talk to my family" (Male young adult, Lima)*

For peer review only

Another helpful resource was the sense of belonging to a group with similar interests and with whom they can share experiences and views. Most frequently reported were sports, artistic and religious groups, but political groups and scouts also came up as helpful resources. These groups helped to forget about problems, openly express feelings, feel understood and supported by peers, and develop skills (i.e., artistic skills). Socialising and communicating in these groups could improve the mood in situations of distress.

V- Social media

Social media were reported as a valuable resource to distract themselves, maintain contact with friends and acquaintances, meet new people or watch inspiring content. However, some participants also referred to negative aspects of social media, such as promoting unrealistic expectations about living standards and physical appearance as well increase the risk of being cyberbullied.

> *"It's a bit 50/50, because it depends on the type of people you have on your social network. Why I say this, because in my case, during school, for a long time I was cyber bullied. I had to close my accounts, report it, everything, all bad. And in the same place I found people who supported me and helped me, motivated me and took me through the path of God. I guess it depends on the type of people you have on your social network and how you use it" (Female young adult, Bogotá)*

VI- Community resources

Community resources were suggested only by participants from Bogotá and Buenos Aires. They included parks, cultural centres, botanical gardens, public libraries and activities such as workshops focused on arts, writing, reading, poetry and sports. Young adults and adolescents highlighted the importance of having access to these spaces to meet other people or engage in activities, which help them forget about their problems.

Professionals stated that these resources and spaces helped young people to develop their identity and build bonds, emphasising more often clubs, schools, and community spaces.

> *"When I go to the cultural centre and dance or when I go to the Scouts on Saturday, I focus so much on that [activity] that I forget about my problems and have a great time" (Female adolescent, Buenos Aires)*

VII- Activities

Arts activities

Arts activities such as playing music, painting, drawing and dance were commonly reported as a resource that allowed participants to meet people with similar interests and express themselves and their emotions freely in a contained context, particularly in situations of discomfort. Such activities

would also help to develop social and problem-solving skills, and gain self-knowledge and confidence. Arts activities were perceived liberating and stress reducing. In addition, professionals stated arts enable young people to explore and develop their talents.

In addition to the above mentioned arts activities, writing was suggested as a resource, mostly by women. Some write stories or poems as it allows them to process and express their emotions, while others prefer free writing about their problems or any other topic as a way to relax and see the problems from another point of view. Other less common artistic activities mentioned were theatre, clown, circus, graffiti, rap, sculpture and photography.

> *"It's not just one thing, not just drawing, not just listening to music, not just writing, it's everything that has to do with art, I feel it liberates me a lot, it gives me peace, calms me down when facing situations so complicated, we have. It is like your own psychologist, to whom you can tell how you feel without thinking of others and that is why I like it so much" (Male young adult, Bogotá)*

Leisure activities

Hobbies and enjoyable leisure activities such as listening to music or attending a concert were reported as helping to manage stress, relax, forget about problems and create supportive bonds with others.

> *"Whenever I felt sad, I listened [to music], I put on my headphones, put some music on that I know I listened to when I felt happy" (Male young adult, Lima)*

Other leisure activities that worked as a welcome distraction from problems and distress were watching television, movies, series, music videos, and broadcasts of sporting events; manual activities such as cooking, knitting, sewing and recycling; playing with pets; reading; listening to podcasts; attending conferences; solving brain teasers; playing games; and going on trips.

Sports activities

A further resource were sports and physical activities which helped to relax, distract, overcome emotional distress and bond with peers.

> *"When I do sports, the problems leave, you focus only on what you have to do or what you want to do, to improve and be okay. It clears your head a lot and that helps you overcome any problem" (Male adolescent, Buenos Aires)*

Outdoor activities

Some participants mentioned outdoor activities as a resource which helped to relax, particularly at times of sadness and distress. These activities included going out for a walk, either alone or in a group, skateboarding and hanging out or sunbathing with friends in the park.

For peer review only

> *"When I have been in those moments [of mental distress] I went to the park and seat there, close my eyes and just listen to the sounds, the wind, how the plants move" (Female young adult, Lima)*

Other outdoor activities were related to contact with nature such as nature walks, going to parks, camping, going to the beach or the riverside.

> *"I noticed watching nature helped me a lot, it relaxes me and helps me process things. To see the sky and more natural environments, I noticed it helps me greatly." (Female young adult, Bogotá)*

VIII- Mental health professionals

As shown in Table 1, more than a third of the young people in the focus groups reported previous experiences with mental health treatment. Their experiences were mostly with psychologists at school, university or youth groups. In Bogotá, some participants also mentioned having participated in support groups and coaching led by psychologists.

The experiences were reported as mostly positive and helpful to solve problems, vent emotions and receive support. Those participants who had never been in contact with a mental health professional, they stated that they would seek contact with a professional only if they had a serious problem, which they could handle on their own.

> *"If the problem has gotten out of my hands, I would go to a psychologist, for guidance" (Male young adult, Lima)*

Discussion

Main findings

Adolescents and young adults living in large cities in Latin America report a range of resources that can help them to recover from mental distress. Across the capitals in three Latin American countries, similar resources were identified. They were grouped into eight categories: personal resources, personal development, spirituality and religion, social resources, community resources, social media, activities (artistic, leisure, sports and outdoor activities), and mental health professionals. Several of the categories overlap, but all of them focus on different types of resources. In their combination they show a rich picture of potential resources that young people from economically disadvantaged districts in large cities in Latin America use and find helpful to cope with mental distress.

The reported resources address personal aspects such as personal development, the expression of emotions, feeling distracted and the acquisition of skills. Most of the resources however have a

For peer review only

strong social element and refer to support received from family, friends and other groups. Various of the most commonly reported activities combine the two aspects: they distract and cheer up the young person, and at the same time they involve being in groups and feeling understood and supported by others.

Strengths and limitations

To our knowledge, this is the first study to explore resources of young people to overcome mental distress in large urban environments across three Latin American countries. Using the same language facilitated the analysis across countries and allowed for direct comparisons. The study captured not only perspectives from three different countries, but also from three groups, i.e. adolescents, young adults and professionals. Moreover, the material was generated through two different methods. We used focus groups as a more conventional form of qualitative approach and structured group conversations embedded in arts workshops which is a more innovative method in this context and provides a different approach to engage young people and encourage them to express their views. Combining the different perspectives and methods yielded comprehensive material with many commonalities, suggesting a validity of the findings.

However, this study also had some limitations. Firstly, and most importantly, focus groups and structured conversations were conducted online. This was inevitable because of the restrictions due to the pandemic. Although this mode of communication posed fewer practical problems than originally expected by the research team and still generated rich material, the online communication may have influenced the interaction between the facilitators and the participants and thus the results. Secondly, the recruitment of a substantial proportion of the participants through arts organisations may have biased the findings, leading to a particularly strong emphasis on arts activities and related group experiences. Thirdly, we conducted the study with young people from economically disadvantaged districts in three large cities in Latin America. The three cities have some commonalities and differences. The cultural, economic and social context in large cities in other Latin American countries may differ so that not all of the findings of this study may be generalisable to cities in those other countries. Fourthly, the study was conducted during the pandemic which may have influenced the views of young people as to what they would find helpful, perhaps emphasising group activities that were difficult to access during that period. Finally, we assessed the views of young people and professionals about helpful resources and have no evidence as to what extent those resources are actually used and are beneficial in reality.

Comparison with existing literature

Several of the findings can be related to other research and appear largely consistent with what has been found in other studies before. Activities related to academic, occupational or vocational development such as work, job placement and entrepreneurship were considered as a helpful

resource to cope with difficult situations and to help young people stay active and motivated. These findings may be related to the lower rates of anxiety and depressive disorders found in adolescents and young adults with higher educational levels in a study in Chile (28).

Receiving support from friends and belonging to a group were considered an important social resource (19). Whilst family was also reported as a helpful resource, this was not as consistently mentioned as one might have expected given the cultural importance of the family in the Latin American context (29). One can only speculate as to whether this was because of the age of the participants who – as adolescents and young adults – were in a phase of life where they take a distance from their family of origin and have not yet founded a new family themselves (30).

Arts activities and sports were two resources frequently reported as helpful to improve mental health. Activities such as painting, dance, music, handcrafts and other creative arts were identified as a form of non-verbal expression of feelings that improves confidence and helps to reduce distress. Several studies have identified the benefits of these types of activities for young people's mental health. A systematic review showed that artistic activities are beneficial as they allow participants to communicate through art, express thoughts and feelings more effectively, increase self-esteem and improve the ability to handle anger (31). Another study showed that teens who take part in weekly extracurricular activities, including art, had less anxiety and depression and were generally more optimistic (32). Similarly, participants suggested that physical activities such as running and team sports were beneficial to reduce stress and improve energy and mood. This finding is consistent with the results of a systematic review showing sports are associated with an improvement in psychosocial health (33).

Implications

The findings may have several implications. For reducing anxiety and depression of an individual or on a group level, not all of the resources listed in this study are likely to be equally available or relevant in a given context However, the eight categories may provide a framework that can help to consider different options for developing programmes that facilitate and strengthen some of the individual and social resources and activities that may help young people to overcome their distress in lower income urban settings.

The findings show that young people come up with a wide range of ideas and experiences about helpful resources. Thus, when considering approaches to improve the mental health of young people, their voice should be heard and take a central role in the development of policies (34). When their views are elicited, innovative methods like arts workshops may help to engage young people and generate helpful material. Based on the experiences in this study, conducting such workshops and interviews online appears a feasible and valuable method to provide a range of findings. Online methods may help to complete similar studies with fewer resources and in shorter

For peer review only

periods of time than studies with conventional in-person interviews, thus making them more feasible, particularly in low-resource settings.

Most of the young people in our study reported to have experienced anxiety and/or depression and more than a third had received some type of treatment, although there is no information about the type of treatment. In any case, young people reported largely positive experiences of mental health professionals. Future policies to improve the mental health of young people in large cities should address the need for accessible and effective mental health services. However, this cannot be the sole focus as the provision of mental health services requires substantial funding and using professionals can come with the implicit message that the affected young person cannot cope on their own, which in turn may undermine confidence rather than strengthen it. On a population level, promoting personal and interpersonal skills at school, support from peers and groups, and access to various activities appear more important to reduce the overall level of mental distress in young people.

Finally, considering that this study was carried out in urban environments, it is important to stress the value of community spaces where young people are able to play sports, meet, engage in enjoyable group activities and distract themselves such as parks, public libraries or theatres. In addition to the accessibility of appropriate space, there should also be community organisations enabling and facilitating different activities, especially since some sports and arts activities require equipment and guidance. Future research may explore to what extent community organisations should be set up and supported to attract, engage and help particularly vulnerable and mentally distressed young people.

Conclusion

Anxiety and depression are frequent among young people. This study shows that in a challenging context like large cities in Latin America, there is a wide range of resources, similar across the three cities, that young people can use and report as helpful. Future research and policies may focus on how these resources can be made widely available, be strengthened and be flexibly utilised by young people if and when required.

Declarations:

Ethics approval and consent to participate:

Written approval was received for this study from the Faculty of Medicine - Research and Ethics Committee of the Pontificia Universidad Javeriana, Bogota on 20/03/2020 (FM-CIE-0241-20) from the Institutional Ethics Committee on Research of the Universidad Peruana Cayetano Heredia, Lima on 31/03/2020 (Constancia E021-03-20), and from the Queen Mary Ethics of Research Committee on 16/11/2020 (QMERC2020/02). All methods were carried out in

For peer review only

accordance with relevant guidelines and regulations. All informed consent/assent was obtained from all subjects and/or their legal guardian(s) for participants aged less than 16 years.

Availability of data and materials:

The consent forms did not specify that the data would be deposited in a public repository, and for this reason we are unable to deposit the data. The de-identified participant dataset will be made available from the corresponding author (Natalia Godoy Casasbuenas, natalia.godoy@javeriana.edu.co) upon reasonable request and subject to a data sharing agreement.

Funding:

This work is supported by the Medical Research Council (grant number: MR/S03580X/1)

Authors' contributions:

SP, LIB, FDC, CGR, JMUR and PH devised the project, the main conceptual ideas and design of the study.

MT, NGC, NO, FC, LHP, CGR, JMUR and FDC participated in data collection during the artistic workshops and focus groups.

MT, NGC, LHP, NO, FC participated in the transcription and data analysis of the structured conversations and focus groups.

MT, NGC, NO wrote the manuscript with input from all authors.

Acknowledgements:

We thank Santiago Lucchetti, Jorge Pasquini, Natalia Ortiz, Karen Ariza and Diliniya Stanislaus Sureshkumar for their contributions in the analysis stage. We thank Victoria Jane Bird for comments on the manuscript.

We would like to acknowledge our arts organisations for their contribution to the OLA programme, Crear Vale La Pena, Fundación Batuta, Familia Ayara, Arenas y Esteras and Teatro La Plaza.

References

1. Somerville LH, Jones RM, Casey BJ. A time of change: behavioral and neural correlates of adolescent sensitivity to appetitive and aversive environmental cues. Brain Cogn. 2010 Feb;72(1):124–33.

2. Spear LP. The adolescent brain and age-related behavioral manifestations. Neurosci Biobehav Rev. 2000 Jun;24(4):417–63.

3. Lopez V, Sanchez K, Killian MO, Eghaneyan BH. Depression screening and education: an examination of mental health literacy and stigma in a sample of Hispanic women [Internet]. Vol. 18, BMC Public Health. 2018. Available from: http://dx.doi.org/10.1186/s12889-018-5516-4

For peer review only

4. van der Heide I, Wang J, Droomers M, Spreeuwenberg P, Rademakers J, Uiters E. The relationship between health, education, and health literacy: results from the Dutch Adult Literacy and Life Skills Survey. J Health Commun. 2013;18 Suppl 1:172–84.

5. Benjet C, Sampson L, Yu S, Kessler RC, Zaslavsky A, Evans-Lacko S, et al. Associations between neighborhood-level violence and individual mental disorders: Results from the World Mental Health surveys in five Latin American cities. Psychiatry Res. 2019 Dec;282:112607.

6. Morgan C, Reininghaus U, Reichenberg A, Frissa S, SELCoH study team, Hotopf M, et al. Adversity, cannabis use and psychotic experiences: evidence of cumulative and synergistic effects. Br J Psychiatry. 2014 Mar 13;204:346–53.

7. World Health Organization. Adolescent mental health. Fact sheet [internet]. 2021 [updated 2021 November 17; cited 2021 November 30]. Available from: https://www.who.int/news-room/fact-sheets/detail/adolescent-mental-health

8. Caqueo-Urízar A, Flores J, Escobar C, Urzúa A, Irarrázaval M. Psychiatric disorders in children and adolescents in a middle-income Latin American country [Internet]. Available from: http://dx.doi.org/10.21203/rs.2.14739/v2

9. Van Ameringen M, Mancini C, Farvolden P. The impact of anxiety disorders on educational achievement. Journal of anxiety disorders. 2003 Jan 1;17(5):561-71.

10. Patel V, Flisher AJ, Hetrick S, McGorry P. Mental health of young people: a global public-health challenge. The Lancet. 2007 Apr 14;369(9569):1302-13.

11. Kim D. Blues from the neighborhood? Neighborhood characteristics and depression. Epidemiologic reviews. 2008 Nov 1;30(1):101-17.

12. Morgan C, Reininghaus U, Reichenberg A, Frissa S, Hotopf M, Hatch SL, SELCoH Study Team. Adversity, cannabis use and psychotic experiences: evidence of cumulative and synergistic effects. The British Journal of Psychiatry. 2014 May;204(5):346-53.

13. Thapar A, Collishaw S, Pine DS, Thapar AK. Depression in adolescence. The Lancet. 2012 Mar 17;379(9820):1056-67.

14. Murcia EH, Losada YL, Rojas LA, Londoño PG. Prevalencia de la depresión infantil en Neiva (Colombia). Avances en Psicología Latinoamericana. 2009 Sep 23;27(1):154-64.

15. Méndez X, Rosa AJ, Montoya M, Espada JP, Olivares J, Sánchez-Meca J. Tratamiento Psicológico de la Depresión Infantil. Psicología conductual. 2002;10(3):563-80.

16. March J, Silva S, Petrycki S, Curry J, Wells K, Fairbank J, et al. Fluoxetine, cognitive-behavioral therapy, and their combination for adolescents with depression: Treatment for Adolescents With Depression Study (TADS) randomized controlled trial. JAMA [Internet]. 2004 Aug 18 [cited 2021 Jul 12];292(7). Available from: https://pubmed.ncbi.nlm.nih.gov/15315995/

For peer review only

17. Stapinski LA, Montgomery AA, Heron J, Jerrim J, Vignoles A, Araya R. Depression symptom trajectories and associated risk factors among adolescents in Chile. PLoS One. 2013 Oct 11;8(10):e78323.

18. Anderson K, Priebe S. Concepts of resilience in adolescent mental health research. Journal of Adolescent Health. 2021 May 24.

19. Ayed N, Toner S, Priebe S. Conceptualizing resilience in adult mental health literature: A systematic review and narrative synthesis. Psychol Psychother. 2019 Sep;92(3):299–341.

20. Zarobe L, Bungay H. The role of arts activities in developing resilience and mental wellbeing in children and young people a rapid review of the literature [Internet]. Vol. 137, Perspectives in Public Health. 2017. p. 337–47. Available from: http://dx.doi.org/10.1177/1757913917712283

21. Hildebrand NA, Celeri EHRV, Morcillo AM, Zanolli M de L. Resilience and mental health problems in children and adolescents who have been victims of violence. Rev Saude Publica. 2019 Jan 31;53:17.

22. Suarez EB. Two decades later: The resilience and post-traumatic responses of Indigenous Quechua girls and adolescents in the aftermath of the Peruvian armed conflict. Child Abuse Negl. 2013 Feb;37(2-3):200–10.

23. Vera-Bachmann D. [Resilience, Poverty and Rurality]. Rev Med Chil. 2015 May;143(5):677–8.

24. Herrera-Moreno D, Carvajal-Ovalle D, Cueva-Nuñez MA, Acevedo C, Riveros-Munévar F, Camacho K, et al. Body image, perceived stress, and resilience in military amputees of the internal armed conflict in Colombia. Int J Psychol Res (Medellin). 2018 Jul;11(2):56–62.

25. Miller-Graff LE, Scheid CR, Guzmán DB, Grein K. Caregiver and family factors promoting child resilience in at-risk families living in Lima, Peru. Child Abuse Negl. 2020 Oct;108:104639.

26. Priebe S, Fung C, Brusco LI, Carbonetti F, Gómez-Restrepo C, Uribe M, Diez-Canseco F, Smuk M, Holt N, Kirkbride JB, Araya R. Which resources help young people to prevent and overcome mental distress in deprived urban areas in Latin America? A protocol for a prospective cohort study. BMJ open. 2021 Sep 1;11(9):e052339.

27. Bengtsson M. How to plan and perform a qualitative study using content analysis. NursingPlus Open. 2016 Jan 1;2:8-14.

28. Gaete J, Labbé N, Del Villar P, Allende C, Araya R, Valenzuela E. Mental health and associated factors among young offenders in Chile: a cross-sectional study. Crim Behav Ment Health. 2018 Feb;28(1):13–27.

29. Kuznesof EA. The House, the Street, Global Society: Latin American Families and Childhood in the Twenty-First Century [Internet]. Vol. 38, Journal of Social History. 2005. p. 859–72. Available from: http://dx.doi.org/10.1353/jsh.2005.0065

30. Parra A, Oliva A, del Carmen Reina M. Family Relationships From Adolescence to Emerging Adulthood [Internet]. Vol. 36, Journal of Family Issues. 2015. p. 2002–20. Available from: http://dx.doi.org/10.1177/0192513x13507570

31. Uttley L, Scope A, Stevenson M, Rawdin A, Taylor Buck E, Sutton A, et al. Systematic review and economic modelling of the clinical effectiveness and cost-effectiveness of art therapy among people with non-psychotic mental health disorders. Health Technol Assess. 2015 Mar;19(18):1–120, v – vi.

32. Thomson LJ, Morse N, Elsden E, Chatterjee HJ. Art, nature and mental health: assessing the biopsychosocial effects of a "creative green prescription" museum programme involving horticulture, artmaking and collections. Perspect Public Health. 2020 Sep;140(5):277–85.

33. Eime RM, Young JA, Harvey JT, Charity MJ, Payne WR. A systematic review of the psychological and social benefits of participation in sport for children and adolescents: informing development of a conceptual model of health through sport. Int J Behav Nutr Phys Act. 2013 Aug 15;10:98.

34. Oxman AD, Lewin S, Lavis JN, Fretheim A. SUPPORT Tools for evidence-informed health Policymaking (STP) 15: Engaging the public in evidence-informed policymaking. Health Res Policy Syst. 2009 Dec 16;7 Suppl 1:S15.

For peer review only

OLA WP1 Arts-based workshops: Topic guide for structured conversations with adolescents and young adults

Objectives: (1) To explore the experiences of emotional and mental health problems, including feelings of depression, anxiety and stress of young people with emphasis on understanding how these episodes have been recovered and overcome. (2) Explore what role they think arts and creativity had or could have had to protect them and help them recover from anxiety and depression.

a. Breaking the ice

What caught your attention in the theatre?
If theatre is removed from your life, what would you lose?

b. Recognizing mental anguish

We want to know what emotional and mental health problems (anxiety / depression / stress) mean for you.

Have you ever experienced it? If not, have you perceived it in people close to you?

How do you experience it? Can you visualize / express it? Can you communicate it to others? Have you found metaphors or symbols or arts to express these feelings?

Have you found any artistic resource to express these feelings, such as workshops, poetry, music, etc?

c. Coping with mental anguish

Where do you go when you feel distressed? Real places? Fantasy worlds? Are you looking for help in your environment? Do you attend reflection groups? Individual therapy? Group therapy? Do you go to Church? Do you play sport or take physical exercise? Or do you take refuge in yourselves? Do you use your imagination? Do you make art?

How do you physically experience anxiety / distress? What other impact does it have on your life?

Who do they talk to about these feelings? Other young people? Friends? Teachers? Families? Where? When? Who can help best in these moments?

Can you map out the resources (people / places / activities) in your daily life that allow you to reduce distressed state and acquire greater strength in triggering situations? What keeps you safe and comfortable? What significant physical and mental changes do you think you have experienced? How could them describe this mood change?

The recovery process is not always linear or progressive, it involves progress and Setbacks. Can you identify what are the situations or elements that influence those setbacks?

What emotions or feelings do you perceive when moving from anxiety to emotional stability?

d. Understanding the relationship between art and resilience

How do the arts and creative practices build your ability to recover from anxiety and distress as well as to develop resilience to mental pathology?

For peer review only

OLA WP1: Topic guide for focus groups with adolescents, young adults and professionals to explore views on resources that young people use to overcome mental distress

Introduction and group rules (5 mins)

Thank participants for their availability, introduce researchers and explain:

- All participants here have signed a consent form that describes the study and the aim of the group.
- Aim of the group: to explore and understand your views on the types of resources that young people use to overcome mental and emotional health problems. By resources we mean what helps people to recover, specifically personal resources (for example, what people do and how people might cope in different situations), and social resources (for example, your friendships).
- We are interested in seeing if the resources that young people use to recover from mental and emotional health problems change as they grow older; and so we are involving young people in our study who are aged 15-16 and 20-24 years.
- Confidentiality: the names of the participants will only be known by the researchers and other participants in today's focus group, and will not be revealed to anyone else.
- The session will be audio-recorded, transcribed and analysed by researchers.
- All participants will be assigned an ID number and all potentially identifying information will be removed.
- Audio recordings will be destroyed once data analysis is complete.
- One researcher will facilitate the discussion using a topic guide. The second researcher will make sure all areas to be discussed are covered and to write notes about important points raised.

<u>Outline group rules</u>:

- Information shared within the group must remain confidential. We ask that you try not to use names if talking about services or staff members.
- Speaking one at a time and listening and respecting others' opinions
- You do not have to share information that you do not want to
- There are no right or wrong answers
- We are looking for the widest possible range of views. Alignment of views within the group is not required
- Mobile phones to be switched off or on silent mode (unless required for work/urgent personal reasons)

- Any questions?
- Start audio recorder

Ice breaker (approx. 5 mins):

Ask each participant in turn to say:

For peer review only

- Their name or nickname
- And something about themselves, e.g. one thing they enjoy doing or a talent they have

Focus group discussion (approx. 45 mins):

Aim: to explore and understand the views and experiences of adolescents, young adults and professionals on the types of resources that young people use, alternative ways of measuring resources and on the resources included in the assessment battery.

During adolescence and youth, it can be common for people to experience mental and emotional health problems, which includes feelings of depression, anxiety and stress. Living in big cities can mean people are more likely to experience stressful events. However, not all people develop mental and emotional health problems, and when people do become emotionally distressed, many will recover within a year. And so we want to understand what helps people to recover.

<u>Introduction:</u>

- <u>What are the situations or experiences that might cause emotional distress in young people?</u>

<u>Types of resources</u>:

- What helps young people to recover from mental and emotional health problems?
- What resources do young people have in their daily life that help with feelings of emotional distress?
- What helps people if they are feeling down, upset or anxious?

Prompts:

Family/partner
Friendships
Groups in community such as religious groups, youth centres
Sports activities – alone or in groups
Arts activities – alone or in groups
Internet/social media
Personal resources (thinking styles, coping strategies, self-care)
Green spaces in cities/going out of the city/contact with nature
Can you think of other things that help people?

- How do they help?

  Prompts:

  Do they help all people, or just some people?
  - Are there resources that are more helpful for young people depending on their gender?
  - What resources are more helpful for young people of male/female/non binary gender?
  - Are there resources that are more helpful for your age group compared to the younger/older age group?

- Are there resources in the local community for young people?

- What type of resources

- How do young people learn about and access resources in the local community?
  - How do people find out about these resources?
  - Do they need a referral to start attending a group, club or organisation?
  - Do they need to pay a fee?

- What are your/young people's experiences of accessing these resources?
  Prompts:
  Was it easy to access them and why?
  Did you face any barriers or difficulties?
- What resources would young people like to see and access in the local community?

<u>Measuring resources</u>:
In this research study, we would like to understand better the resources that help young people to overcome mental and emotional health problems. To help with this, we have put together a booklet to ask young people about the resources that they use. The booklet consists of a range of questionnaires that can be completed by young people. At the moment, the resources that are included in the booklet are: (show and read through list of resources).

- In your opinions, are there resources or areas that are important for mental and emotional health to young people, which are missing from the booklet?
  - What resources?
  - Why?
  - How would you ask young people about these resources?

- Are there resources in the booklet, which you think are not relevant for young people?
  - What resources?
  - Why?

- Do you have any other thoughts or comments about the resources in the booklet?

- We currently plan to ask young people about these resources using a booklet of questionnaires. What do you think about this approach?
  - How should young people complete this booklet of questionnaires? (by themselves, with the help of a researcher?)

- What other ways do you think we could use to identify and ask about the resources that young people use, apart from this booklet?
  - Can you think of other approaches to explore resources with young people?

Conclusion (up to 10 mins)

For peer review only

- Does anyone have anything else to add to what has been discussed today: any further comments or recommendations about resources for recovering from mental and emotional health problems?
- Does anyone have questions for us about this study?
- Thank participants for their contribution

For peer review only

Reflexivity Statement

1. How does this study address local research and policy priorities?

The study explores which personal and social resources young people for deprived neighbourhoods in large Latin American cities use to overcome episodes of anxiety and depression. Growing up in deprived neighbourhoods is associated with a number of risk factors for developing mental disorders such as poverty, violence and social inequality. Anxiety and depression are frequent among young people in those neighbourhoods, but evidence suggests that about 50% of affected people overcome their distress within a year. The study explored which resources they use in that process. The mental health of young people is a societal priority across the world, and the challenge of improving it has become even more difficult during the pandemic. Since not all young people can or should access professional health services for help, a good understanding of what resources they use and find helpful is essential. Thus, the study addresses an area of utmost relevance for local policies and health.

2. How were local researchers involved in study design?

The study was developed in a group process with researchers from Bogotá, Buenos Aires and Lima as equal partners. Most decisions were taken in a meeting in Lima in 2019 where consensus was reached on the study design. Since the study was conducted in Bogotá, Buenos Aires and Lima, the views of the local researchers were central to the design of the study and to its organisation, and they also led on collaborating with local arts organisations.

3. How has funding been used to support the local research team?

Most of the funding went to the local research teams and their activities on the study were fully funded by the grant. How precisely they used the funding in terms of contracts with research assistants and support for activities was the decision of local teams and varied across the participating cities.

4. How are research staff who conducted data collection acknowledged?

There are three co-authors from each partner, who led on the data collection. In the acknowledgement we also mention all the research assistants who were involved in the data collection although their role did not meet the criteria for authorship.

5. Do all members of the research partnership have access to study data?

Yes.

6. How was data used to develop analytical skills within the partnership?

The data was analysed by the local research teams. Since the material is in Spanish, only they were able to analyse all the data in full. They conducted the analysis and drafted the first version of the paper, involving researchers from all three partner cities. Subsequently, the leading authors are from the partners in Latin America.

7. How have research partners collaborated in interpreting study data?

For peer review only

They led on the interpretation of the data and wrote the first version of the paper, with the team in London providing support.

8. How were research partners supported to develop writing skills?

The paper was written by the research partners and then discussed, further developed and improved in an iterative communication with the Chief Investigator from London. Thus, the researchers leading on the paper in the partner countries received on-going feed-back and were involved in all steps of the editing of the paper.

9. How will research products be shared to address local needs?

We believe that some of the results are of policy relevance to improve the mental health of young people in deprived areas in Latin American cities. Once published, we will disseminate the findings further using social media, personal communications, presentations and links of the partners with local policy makers.

10. How is the leadership, contribution and ownership of this work by LMIC researchers recognised within the authorship?

There are 12 authors in total. First authorship is shared between the leading author from Colombia and Peru with the leading research from Argentina being the third author. There are two further researchers from each partner as authors (in alphabetical order). The only authors not from the partners are the co-ordinator of the participating arts organisations across Latin America (PH), the programme manager supporting all activities and co-ordinating communication across sites throughout the study (CF), and the Chief Investigator of the study who provided guidance and support throughout the process, including the paper writing stage.

11. **How have early career researchers across the partnership been included within the authorship team?**

There are five early career researchers among the authors, including the three leading authors (MT, NGC, NO).

12. How has gender balance been addressed within the authorship?

First authorship is shared between a female and a male researcher. The corresponding author is female. The third author leading for Argentina is female. Overall, there are eight men and four women among the authors.

13. How has the project contributed to training of LMIC researchers?

The study is part of a research programme with a strong emphasis on the training and promotion of early career researchers in Latin America. The funding provides them with the possibility to focus on research and develop their skills in research. Various components of the programme contributed that which includes specific seminars on critical appraisal and paper writing.

14. How has the project contributed to improvements in local infrastructure?

The significant funding of the research programme has established an infrastructure for mental health research that had not existed in the same way before. The exact context and set up vary

For peer review only

across partners. Whilst all partners aim to make the infrastructure sustainable, it remains to be seen how much of the new infrastructure can be maintained beyond the end of the research programme.

15. What safeguarding procedures were used to protect local study participants and researchers?

The safeguarding procedures met international standards and were specified in the applications to the ethics committee in each country and in London.

For peer review only

BMJ Open

Identifying resources used by young people to overcome mental distress in three Latin American cities: A qualitative study

Journal:	*BMJ Open*
Manuscript ID	bmjopen-2021-060340.R1
Article Type:	Original research
Date Submitted by the Author:	14-Apr-2022
Complete List of Authors:	Toyama, Mauricio; Universidad Peruana Cayetano Heredia, CRONICAS Center of Excellence in Chronic Diseases<br>Godoy-Casasbuenas, Natalia; Pontificia Universidad Javeriana, Department of Clinical Epidemiology and Biostatistics<br>Olivar, Natividad; Universidad de Buenos Aires, Department of Psychiatry and Mental Health, School of Medicine<br>Brusco, Luis Ignacio; Universidad de Buenos Aires, Department of Psychiatry and Mental Health, School of Medicine<br>Carbonetti, Fernando; Universidad de Buenos Aires, Department of Psychiatry and Mental Health, School of Medicine<br>Diez-Canseco, Francisco; Universidad Peruana Cayetano Heredia, CRONICAS Center of Excellence in Chronic Diseases<br>Gómez-Restrepo, Carlos; Pontificia Universidad Javeriana, Department of Clinical Epidemiology and Biostatistics; Pontificia Universidad Javeriana, Department of Psychiatry and Mental Health<br>Heritage, Paul; Queen Mary University of London School of English and Drama<br>Hidalgo-Padilla, Liliana; Universidad Peruana Cayetano Heredia, CRONICAS Center of Excellence in Chronic Diseases<br>Uribe, Miguel ; Pontificia Universidad Javeriana, Department of Psychiatry and Mental Health<br>Fung, Catherine ; Queen Mary University of London, Unit for Social and Community Psychiatry<br>Priebe, Stefan; Queen Mary University of London, Unit for Social and Community Psychiatry
**Primary Subject Heading**:	Mental health
Secondary Subject Heading:	Qualitative research, Public health, Global health
Keywords:	MENTAL HEALTH, QUALITATIVE RESEARCH, Child & adolescent psychiatry < PSYCHIATRY, Depression & mood disorders < PSYCHIATRY

SCHOLARONE™
Manuscripts

I, the Submitting Author has the right to grant and does grant on behalf of all authors of the Work (as defined in the below author licence), an exclusive licence and/or a non-exclusive licence for contributions from authors who are: i) UK Crown employees; ii) where BMJ has agreed a CC-BY licence shall apply, and/or iii) in accordance with the terms applicable for US Federal Government officers or employees acting as part of their official duties; on a worldwide, perpetual, irrevocable, royalty-free basis to BMJ Publishing Group Ltd ("BMJ") its licensees and where the relevant Journal is co-owned by BMJ to the co-owners of the Journal, to publish the Work in this journal and any other BMJ products and to exploit all rights, as set out in our licence.

The Submitting Author accepts and understands that any supply made under these terms is made by BMJ to the Submitting Author unless you are acting as an employee on behalf of your employer or a postgraduate student of an affiliated institution which is paying any applicable article publishing charge ("APC") for Open Access articles. Where the Submitting Author wishes to make the Work available on an Open Access basis (and intends to pay the relevant APC), the terms of reuse of such Open Access shall be governed by a Creative Commons licence – details of these licences and which Creative Commons licence will apply to this Work are set out in our licence referred to above.

Other than as permitted in any relevant BMJ Author's Self Archiving Policies, I confirm this Work has not been accepted for publication elsewhere, is not being considered for publication elsewhere and does not duplicate material already published. I confirm all authors consent to publication of this Work and authorise the granting of this licence.

For peer review only

Title: Identifying resources used by young people to overcome mental distress in three Latin American cities: A qualitative study

Corresponding author:
Natalia Godoy-Casasbuenas
Postal address: Carrera 7ª # 40-62, 2nd floor – Bogotá, Colombia
E-mail: natalia.godoy@javeriana.edu.co
Telephone: (+571) 3208320 ext. 2758

Authors:
Mauricio Toyama[1], Natalia Godoy-Casasbuenas[2], Natividad Olivar[3], Luis Ignacio Brusco[3], Fernando Carbonetti[3], Francisco Diez-Canseco[1], Carlos Gómez-Restrepo[2,4], Paul Heritage[5], Liliana Hidalgo-Padilla[1], José Miguel Uribe-Restrepo[4], Catherine Fung[6], Stefan Priebe[6]

Authors information:
[1]CRONICAS Centre of Excellence in Chronic Diseases, Universidad Peruana Cayetano Heredia, Lima, Perú

[2]Department of Clinical Epidemiology and Biostatistics, Pontificia Universidad Javeriana, Bogotá, Colombia

[3]Department of Psychiatry and Mental Health, School of Medicine, Universidad de Buenos Aires, Buenos Aires, Argentina

[4]Department of Psychiatry and Mental Health, Pontificia Universidad Javeriana, Bogotá, Colombia

[5]School of English and Drama, Queen Mary University of London, London, United Kingdom

[6]Unit for Social and Community Psychiatry (WHO Collaborating Centre for Mental Health Service Development), Queen Mary University of London, London, United Kingdom

Key words: Mental Health, Adolescent psychiatry, Depression, Anxiety, Qualitative Research

Abstract count: 295
Word count: 5312

Number of references: 36

For peer review only

Abstract (295 words):

Objective: To explore which resources and activities help young people living in deprived urban environments in Latin America to recover from depression and/or anxiety.

Design: Multimethod, qualitative study with 18 online focus groups and 12 online structured group conversations embedded into arts workshops.

Setting: This study was conducted in Bogotá (Colombia), Buenos Aires (Argentina) and Lima (Peru)

Participants: Adolescents (15-16 years old) and young adults (20-24 years old) with capacity to provide assent/consent; and professionals (older than 18 years of age) that had experience of professionally working with young people, were willing to share personal experience within a group, and had capacity to provide consent.

Results: A total of 185 participants took part in this study: 111 participants (36 adolescents, 35 young adults and 40 professionals) attended the 18 focus groups; and 74 young people (29 adolescents and 45 young adults) took part in the 12 arts workshops. Eight categories captured the resources and activities that were reported by young people as helpful to overcome mental distress: i) Personal resources, ii) Personal development, iii) Spirituality and religion, iv) Social resources, v) Social media, vi) Community resources, vii) Activities (sub-categorized into artistic, leisure, sports and outdoor activities) and viii) Mental health professionals. Personal and social resources as well as artistic activities and sports were the most common resources identified that help adolescents and young adults to overcome depression and anxiety.

Conclusion: Despite the different contexts of the three cities, young people appear to use similar resources to overcome mental distress. Policies to improve the mental health of young people in deprived urban settings should address the need of community spaces where young people can play sports, meet, and engage in groups, and support community organisations that can enable and facilitate a range of social activities.

Strengths and limitations

- This is the first study to explore resources of young people to overcome mental distress in large urban environments across three Latin American countries.
- The study combined different perspectives (adolescents, young adults, professionals) and methods (focus groups, structured group conversations embedded in arts workshops) providing a different approach to engage young people and encourage them to express their views.

For peer review only

For peer review only - http://bmjopen.bmj.com/site/about/guidelines.xhtml

- The recruitment of a substantial proportion of participants was done through arts organisations which may have biased the findings, leading to a particularly strong emphasis on arts activities.
- The study was conducted during the Covid-19 pandemic which may have influenced the views of young people as to what they find helpful.
- Focus groups and structured group conversations were conducted online, which may have influenced the interaction between the facilitators and the participants and thus the results.

For peer review only

For peer review only - http://bmjopen.bmj.com/site/about/guidelines.xhtml

Introduction

Adolescence and young adulthood represent a period of growth and development. Young people experience physical changes as well as changes in their cognitions, emotions and social environment (1,2). At the same time, cultural, socioeconomic, and environmental influences, including poverty, low education (3,4), violence (5), or substance use (6), make adolescents and young adults particularly vulnerable to developing mental health problems.

According to the World Health Organization (WHO), it is estimated that between 10% and 20% of young people experience mental disorders, most commonly depression and anxiety disorders (7). Depression represents a common of illness and disability in this group, can be associated with substance abuse, aggressiveness, eating disorders, and is one of the main risk factors for suicide. Anxiety can be accompanied by disturbances of sleep, concentration, social and/or occupational functioning (8), and is the ninth cause of illness and disability in this population (9). Poor mental health at young age is associated with further health and social problems such as poorer academic achievement, early school drop-out and substance abuse (10).

Young people living in major cities across Latin America can be exposed to various risk factors for developing mental disorders, such as poverty, violence and social inequality (11-14). The percentage of young people with depression and/or anxiety has been reported to range from 17% in Colombia (15) to 26% in Argentina (16). However, longitudinal studies have found that among young people who suffer from depression and anxiety, between 50 and 60% manage to overcome their distress within one year (17,18).

The ability to overcome mental distress – sometimes referred to as resilience - is linked to personal resources such as motivation and hope and to social resources such as support from peers and access to activities in the surrounding environment (19, 20). In children and young adults, arts activities can be an important social resource (21). In Latin America, studies about resources to cope with adversity have been carried out in the context of social violence (22,23), poverty (24), forced displacement (25) and medical conditions (26), but to our knowledge no study has explored the resources that help young people in this region to overcome mental distress.

The aim of this study is to explore the participant´s perceptions of which resources and activities help young people in large cities in Latin America to recover from mental distress in form of depression and/or anxiety, using both conventional focus groups and arts workshops.

Methods

Study design

This is an exploratory qualitative study using a grounded theory design (27). The study was conducted within the context of a larger research programme called "Building resilience and

For peer review only - http://bmjopen.bmj.com/site/about/guidelines.xhtml

resources to reduce depression and anxiety in young people from urban neighbourhoods in Latin America (OLA)". The overall aim of the programme is to identify which characteristics, resources and activities help young people living in urban environments in Latin America to prevent or recover from depression and/or anxiety (28). The programme is a collaboration between Queen Mary, University of London (UK), Universidad de Buenos Aires (Argentina), Pontificia Universidad Javeriana in Bogotá (Colombia) and Universidad Peruana Cayetano Heredia in Lima (Peru). The researchers work in close partnerships with the following arts organisations: Crear Vale La Pena (Argentina), Fundación Batuta and Familia Ayara (Colombia) and Teatro La Plaza (Peru). These organisations specialize in building creative environments, using classical music, rap music, street art, dance, theatre and visual arts, respectively.

This study used two forms of group activities for data collection: focus groups including adolescents (15/16 years), young adults (20-24 years) and professionals working with young people; and arts workshops with young people (15-24 years). The arts workshops were intended as a different approach to engage young people and elicit their views. Towards the end or following the arts activities, all arts workshops included a structured group conversation to explore resources to overcome anxiety and/or depression. Because of the restrictions due to the pandemic, all groups were conducted online between August and November of 2020.

A total of 18 focus groups were conducted: six in each country, two for each group of participants (adolescents, young adults and professionals). The focus groups lasted between approximately 90 and 120 minutes. There were also 12 arts workshops: four in Argentina using a combination of creative activities run by the arts organisation 'Crear Vale La Pena'; two in Colombia using rap and graffiti run by 'Familia Ayara' and four with musical activities run by 'Fundación Batuta'; two in Peru using theatre and dramaturgical exercises run by 'Teatro La Plaza'. The length of the workshops varied. They usually lasted between two and three hours. Each focus group and arts workshop had a facilitator and a co-facilitator or technical supporter.

Study setting

The study was conducted in the capital cities of three Latin American countries - Buenos Aires (Argentina), Bogotá (Colombia) and Lima (Peru) – and focused on young people from poorer economic neighbourhoods based on national indicators of income.

Participants

Three groups of participants were included in the study: adolescents, young adults and professionals with experience in working with young people.

For the focus groups, the inclusion criteria were: 15-16 years or 20-24 years of age, respectively; willingness to share personal experiences within the group; capacity to provide assent/consent; and, for the adolescents, consent from a parent/legal guardian. Professionals were included if they

For peer review only - http://bmjopen.bmj.com/site/about/guidelines.xhtml

were at least 18 years of age, had experience of professionally working with young people, were willing to share personal experience within a group, and had capacity to provide consent.

Inclusion criteria for the arts workshops were: 15-24 years of age, current or past participation in activities of the collaborating arts organisations, capacity to provide assent/consent, and, for those under 18 years old, consent from a parent/legal guardian.

Data collection tools

Basic sociodemographic data and previous experience of anxiety and/or depression were collected from all participants in the focus groups on a brief questionnaire, completed by phone or through videoconference (e.g., Zoom).

Topic guides for both focus groups and structured group conversations within the arts workshops addressed the resources used by young people when experiencing distress in form of anxiety and/or depression (See supplementary files 1 and 2). The same topic guides were applied in the three countries.

Procedures

Participants were identified through schools, health services, non-governmental organisations, and youth organisations as well as the four participating arts organisations. Potentially interested participants were contacted via phone by the research team to check the inclusion criteria before they were invited to formally participate in the study. The informed consent procedures were conducted through phone or videoconference. Participants were given the options to provide their consent by phone recording, completing the consent form virtually or completing the form on paper and sending a scan or picture to the research team. Adolescents provided their informed assent, and a parent or legal guardian was contacted to receive their permission and consent. The focus groups and structured group conversations were conducted by researchers trained in qualitative data collection and analysis.

Data analysis

Audio recordings of the focus groups and structured group conversations were transcribed verbatim. We conducted an inductive content analysis using the four stages proposed by Bengtsson (29). The research team from each country analysed the transcriptions of their local participants. At least two members of the research teams in each country worked together during the analysis. During the first stage, decontextualization, the research teams familiarised themselves with the data and inductively created codes based on the relevant information they identified. These codes were added to a spreadsheet shared between the teams. During the recontextualization stage, the codes and categories were discussed in weekly meetings to standardize the codes across the three countries to ensure reliability. After the meetings, each team revisited their transcriptions to either

For peer review only - http://bmjopen.bmj.com/site/about/guidelines.xhtml

re-code their coding to match those identified in other countries, or to code new information they may have missed, based on codes created by the other teams. Furthermore, during the categorisation stage, the codes were discussed and grouped into categories based on similarity of content. These categories were standardized across the research teams. Finally, during the compilation stage, each team conducted a final analysis, summarizing the information extracted for each code in a table. This table was further summarised in a second round to reduce the amount of information. Subsequently, these summaries were combined into a summary encompassing the information from all three countries.

Patient and Public Involvement

Adolescents and young adults (aged 15-24 years old) with knowledge and lived experience of mental distress, including depression and anxiety, were included as part of the Lived Experience Advisory Panel (LEAP) in the three cities where the study was conducted (Buenos Aires, Bogotá, Lima). We received input from the LEAP committee during the design and conduct of the study. The main roles of the LEAP were to provide expert advice to the local research team on all appropriate aspects of the project, including research materials, methods, results, and dissemination.

Ethical statement

The study protocol and all procedures were approved by the Institutional Review Board (IRB) of Universidad de Buenos Aires on February 10th 2020, Pontificia Universidad Javeriana on March 3rd 2020, and Universidad Peruana Cayetano Heredia on March 31st 2020. Furthermore, it was approved by the IRB of Queen Mary University of London on June 5th 2020.

Results

Participants' characteristics

A total of 185 participants took part in this study: 111 participants (36 adolescents, 35 young adults and 40 professionals) attended the 18 focus groups, on average approximately six participants per group in each country; and 74 young people (29 adolescents and 45 young adults) took part in the 12 arts workshops. The average number of participants in the workshops varied across the three countries with five in Argentina, six in Colombia and nine in Peru.

The socio-demographic characteristics of all groups are shown for each city in Tables 1 and 2.

Table 1. Socio-demographic characteristics of young people in focus groups and arts workshops

Focus Groups				
	**Buenos Aires**	**Bogotá**	**Lima**	**Total**

**Age group**				
Adolescents	13 (48%)	9 (50%)	14 (54%)	36 (51%)
Young adults	14 (52%)	9 (50%)	12 (46%)	35 (49%)
**Education level completed**				
Primary school	17 (63%)	9 (50%)	14 (54%)	40 (56%)
Secondary school	4 (15%)	6 (33%)	9 (35%)	19 (27%)
Higher/technical education	6 (22%)	-	3 (12%)	9 (13%)
Other	-	3 (17%)	-	3 (4%)
**Previous experience of anxiety and/or depression**				
Yes	17 (63%)	14 (78%)	18 (69%)	49 (69%)
No	10 (37%)	4 (22%)	8 (31%)	22 (31%)
**Previous experiences with mental health treatment of those who reported previous experience of anxiety and/or depression**				
Yes	9 (53%)	11 (79%)	7 (39%)	27 (55%)
No	8 (47%)	3 (21%)	11 (61%)	22 (45%)
**Arts workshops**				
**Age group**				
Adolescents	9 (45%)	11 (30%)	9 (53%)	29 (39%)
Young adults	11 (55%)	26 (70%)	8 (47%)	45 (61%)

Table 2. Socio-demographic characteristics of professionals participating in focus groups

	**Buenos Aires**	**Bogotá**	**Lima**	**Total**
Psychologists	4 (29%)	4 (29%)	3 (25%)	11 (28%)
Teachers	3 (21%)	6 (43%)	1 (8%)	10 (25%)
Youth workers	3 (21%)	2 (14%)	3 (25%)	8 (20%)
Psychiatrists	2 (14%)	-	-	2 (5%)
Other (e.g.,	2 (14%)	2 (14%)	5 (42%)	7 (23%)

For peer review only - http://bmjopen.bmj.com/site/about/guidelines.xhtml

volunteer, researcher, consultant)				

Resources identified

The resources and activities that help young people to recover from mental distress were grouped into 8 categories: I- Personal resources, II- Personal development, III- Spirituality and religion, IV- Social resources, V- Social media, VI- Community resources, VII- Activities (subcategorised into artistic, leisure, sports and outdoor activities), and VIII- Mental health professionals. Due to the large amount of data collected, only the most commonly reported resources are presented here.

I - Personal resources

Young people, adolescents and professionals refer to personal resources as tools to overcome mental health problems. These resources can be allocated to four sub-categories: relationship with oneself, personal traits, coping strategies and passive strategies.

The first sub-category, relationship with oneself, emphasised the importance of self-awareness and self-esteem. Some participants mentioned that having clear goals builds their confidence and helps them overcome problems.

> *"It is very important to value yourself and accept the problem and know how to handle the situation (...) It is also very important to know yourself and know what you like and do not like to know what can help you with the distress" (Female adolescent, Buenos Aires)*

The second subcategory, personal traits, refers to what young people consider to be the key in overcoming mental distress. Most commonly, empathy was mentioned as a helpful trait, but also resilience and motivation.

The third subcategory were coping strategies, including reflecting about problems, accepting and thinking about a difficult situation, controlling emotions and talking to oneself.

The last subcategory 'passive strategies' involved avoiding or isolating themselves from other people when feeling distressed.

> *"When I feel depressed or sad, I make an abrupt change to the situation, I stop what I am doing, I leave and, for instance, listen to music, draw, or, since generally I do not go out, only sometimes, I prefer to be alone, it is how I feel more peaceful" (Male young adult, Bogotá)*

For peer review only - http://bmjopen.bmj.com/site/about/guidelines.xhtml

II- Personal development

Personal development comprises activities related to academic, occupational and/or vocational development. Young adults mentioned work, job placement and entrepreneurship as a helpful resource to cope with difficult situations and stay active and motivated. School attendance was perceived by professionals as a protective factor for adolescents.

> *"Young people without access to school, or outside educational activities, without access to a recreational space, are negatively affected. Economic factors influence a lot and a lack of resources is closely related to the economic situation" (Psychologist, Buenos Aires)*

III- Spirituality and religion

Religion and spirituality were reported as important resources by participants from Bogotá and Lima. For spirituality, some participants from Bogotá stated that meditation as a practice helped them to relax, calm their mind and get rid of negative energy. Some participants from Lima, mostly young women, mentioned exercises related to relaxation, such as breathing exercises, yoga, meditation, and mindfulness.

Religion was suggested as a resource by participants from both cities. In Bogotá, religion was seen as a resource in moments of sadness and depression and a way to seek guidance for solving problems. In Lima, some participants talked about praying or reading the bible and talking to God was regarded in a similar manner as meditating.

> *"I am Christian, but it's not like I go to confession with a priest (laughs). It's more like simply talking with God, I tell Him 'Father, this happened to me'. It's curious because I did not understand very well from a scientific point of view why that happened, but there is research that says if you are talking with someone, even if they don't exist, it is like a direct meditation. That's how I see it." (female, adolescent, Lima)*

Some participants from Bogotá pointed out that religion can make people feel judged or not good enough. Some participants from Bogotá and Lima remarked that young people are not as religious as older people.

IV- Social resources

Social resources focused on receiving support from friends, family and other people, as well as participating in group activities. Participants from all three cities agreed that support from friends is an important source of emotional support for young people. Talking about their problems made them feel better and engaging in activities with friends was a welcome distraction. Professionals highlighted friends and social groups as an important resource, in some cases more important than the family.

For peer review only

"I believe it is fundamental, the support, the emotional support that family or friends can provide. Like someone mentioned, a lot of the time you confide in your friends more than in your family" (Male young adult, Bogotá)

Repeatedly, young people referred to the family as an important source of support, providing helpful advice and making them feel calmer and more relaxed. Family support was seen as a valuable resource to navigate situations of emotional distress. Other social sources of support were partners and teachers.

"In my case, I talk more with my family, my mom, my sisters, my dad, mostly about things that happen at work, I always talk to them. First, because they are closer to me and because I believe they have the experience to give me advice, with other issues, maybe outside of work, I may talk to friends, but almost always I talk to my family" (Male young adult, Lima)

Another helpful resource was the sense of belonging to a group with similar interests and with whom they can share experiences and views. Most frequently reported were sports, artistic and religious groups, but political groups and scouts also came up as helpful resources. These groups helped to forget about problems, openly express feelings, feel understood and supported by peers, and develop skills (i.e., artistic skills). Socialising and communicating in these groups could improve the mood in situations of distress.

V- Social media

Social media were reported as a valuable resource to distract themselves, maintain contact with friends and acquaintances, meet new people or watch inspiring content. However, some participants also referred to negative aspects of social media, such as promoting unrealistic expectations about living standards and physical appearance as well increase the risk of being cyberbullied.

"It's a bit 50/50, because it depends on the type of people you have on your social network. Why I say this, because in my case, during school, for a long time I was cyber bullied. I had to close my accounts, report it, everything, all bad. And in the same place I found people who supported me and helped me, motivated me and took me through the path of God. I guess it depends on the type of people you have on your social network and how you use it" (Female young adult, Bogotá)

VI- Community resources

Community resources were suggested only by participants from Bogotá and Buenos Aires. They included parks, cultural centres, botanical gardens, public libraries and activities such as workshops focused on arts, writing, reading, poetry and sports. Young adults and adolescents highlighted the importance of having access to these spaces to meet other people or engage in activities, which help them forget about their problems.

For peer review only - http://bmjopen.bmj.com/site/about/guidelines.xhtml

Professionals stated that these resources and spaces helped young people to develop their identity and build bonds, emphasising more often clubs, schools, and community spaces.

> *"When I go to the cultural centre and dance or when I go to the Scouts on Saturday, I focus so much on that [activity] that I forget about my problems and have a great time" (Female adolescent, Buenos Aires)*

VII- Activities

Arts activities

Arts activities such as playing music, painting, drawing and dance were commonly reported as a resource that allowed participants to meet people with similar interests and express themselves and their emotions freely in a contained context, particularly in situations of discomfort. Such activities would also help to develop social and problem-solving skills, and gain self-knowledge and confidence. Arts activities were perceived liberating and stress reducing. In addition, professionals stated arts enable young people to explore and develop their talents.

In addition to the above mentioned arts activities, writing was suggested as a resource, mostly by women. Some write stories or poems as it allows them to process and express their emotions, while others prefer free writing about their problems or any other topic as a way to relax and see the problems from another point of view. Other less common artistic activities mentioned were theatre, clown, circus, graffiti, rap, sculpture and photography.

> *"It's not just one thing, not just drawing, not just listening to music, not just writing, it's everything that has to do with art, I feel it liberates me a lot, it gives me peace, calms me down when facing situations so complicated, we have. It is like your own psychologist, to whom you can tell how you feel without thinking of others and that is why I like it so much" (Male young adult, Bogotá)*

Leisure activities

Hobbies and enjoyable leisure activities such as listening to music or attending a concert were reported as helping to manage stress, relax, forget about problems and create supportive bonds with others.

> *"Whenever I felt sad, I listened [to music], I put on my headphones, put some music on that I know I listened to when I felt happy" (Male young adult, Lima)*

Other leisure activities that worked as a welcome distraction from problems and distress were watching television, movies, series, music videos, and broadcasts of sporting events; manual activities such as cooking, knitting, sewing and recycling; playing with pets; reading; listening to podcasts; attending conferences; solving brain teasers; playing games; and going on trips.

For peer review only - http://bmjopen.bmj.com/site/about/guidelines.xhtml

Sports activities

A further resource were sports and physical activities which helped to relax, distract, overcome emotional distress and bond with peers.

> *"When I do sports, the problems leave, you focus only on what you have to do or what you want to do, to improve and be okay. It clears your head a lot and that helps you overcome any problem" (Male adolescent, Buenos Aires)*

Outdoor activities

Some participants mentioned outdoor activities as a resource which helped to relax, particularly at times of sadness and distress. These activities included going out for a walk, either alone or in a group, skateboarding and hanging out or sunbathing with friends in the park.

> *"When I have been in those moments [of mental distress] I went to the park and seat there, close my eyes and just listen to the sounds, the wind, how the plants move" (Female young adult, Lima)*

Other outdoor activities were related to contact with nature such as nature walks, going to parks, camping, going to the beach or the riverside.

> *"I noticed watching nature helped me a lot, it relaxes me and helps me process things. To see the sky and more natural environments, I noticed it helps me greatly." (Female young adult, Bogotá)*

VIII- Mental health professionals

As shown in Table 1, more than a third of the young people in the focus groups reported previous experiences with mental health treatment. Their experiences were mostly with psychologists at school, university or youth groups. In Bogotá, some participants also mentioned having participated in support groups and coaching led by psychologists.

The experiences were reported as mostly positive and helpful to solve problems, vent emotions and receive support. Those participants who had never been in contact with a mental health professional, they stated that they would seek contact with a professional only if they had a serious problem, which they could handle on their own.

> *"If the problem has gotten out of my hands, I would go to a psychologist, for guidance" (Male young adult, Lima)*

Discussion

Main findings

For peer review only - http://bmjopen.bmj.com/site/about/guidelines.xhtml

Adolescents and young adults living in large cities in Latin America report a range of resources that can help them to recover from mental distress. Across the capitals in three Latin American countries, similar resources were identified. They were grouped into eight categories: personal resources, personal development, spirituality and religion, social resources, community resources, social media, activities (artistic, leisure, sports and outdoor activities), and mental health professionals. Several of the categories overlap, but all of them focus on different types of resources. In their combination they show a rich picture of potential resources that young people from economically disadvantaged districts in large cities in Latin America use and find helpful to cope with mental distress.

The reported resources address personal aspects such as personal development, the expression of emotions, feeling distracted and the acquisition of skills. Most of the resources however have a strong social element and refer to support received from family, friends and other groups. Various of the most commonly reported activities combine the two aspects: they distract and cheer up the young person, and at the same time they involve being in groups and feeling understood and supported by others.

Strengths and limitations

To our knowledge, this is the first study to explore resources of young people to overcome mental distress in large urban environments across three Latin American countries. Using the same language facilitated the analysis across countries and allowed for direct comparisons. The study captured not only perspectives from three different countries, but also from three groups, i.e. adolescents, young adults and professionals. Moreover, the material was generated through two different methods. We used focus groups as a more conventional form of qualitative approach and structured group conversations embedded in arts workshops which is a more innovative method in this context and provides a different approach to engage young people and encourage them to express their views. Combining the different perspectives and methods yielded comprehensive material with many commonalities, suggesting a validity of the findings.

However, this study also had some limitations. Firstly, and most importantly, focus groups and structured group conversations were conducted online. This was inevitable because of the restrictions due to the pandemic. Although this mode of communication posed fewer practical problems than originally expected by the research team and still generated rich material, the online communication may have influenced the interaction between the facilitators and the participants and thus the results. Secondly, the recruitment of a substantial proportion of the participants through arts organisations may have biased the findings, leading to a particularly strong emphasis on arts activities and related group experiences. Thirdly, we conducted the study with young people from economically disadvantaged districts in three large cities in Latin America. The three cities have some commonalities and differences. The cultural, economic and social context in large

For peer review only - http://bmjopen.bmj.com/site/about/guidelines.xhtml

cities in other Latin American countries may differ so that not all of the findings of this study may be generalisable to cities in those other countries. Fourthly, the study was conducted during the pandemic which may have influenced the views of young people as to what they would find helpful, perhaps emphasising group activities that were difficult to access during that period. Finally, we assessed the views of young people and professionals about helpful resources which may not be representative of the experience of all young people in Latin America and have no evidence as to what extent those resources are actually used and are beneficial in reality.

Comparison with existing literature

Several of the findings can be related to other research and appear largely consistent with what has been found in other studies before. Activities related to academic, occupational or vocational development such as work, job placement and entrepreneurship were considered as a helpful resource to cope with difficult situations and to help young people stay active and motivated. These findings may be related to the lower rates of anxiety and depressive disorders found in adolescents and young adults with higher educational levels in a study in Chile (30).

Receiving support from friends and belonging to a group were considered an important social resource (20). Whilst family was also reported as a helpful resource, this was not as consistently mentioned as one might have expected given the cultural importance of the family in the Latin American context (31). One can only speculate as to whether this was because of the age of the participants who – as adolescents and young adults – were in a phase of life where they take a distance from their family of origin and have not yet founded a new family themselves (32).

Arts activities and sports were two resources frequently reported as helpful to improve mental health. Activities such as painting, dance, music, handcrafts and other creative arts were identified as a form of non-verbal expression of feelings that improves confidence and helps to reduce distress. Several studies have identified the benefits of these types of activities for young people's mental health. A systematic review showed that artistic activities are beneficial as they allow participants to communicate through art, express thoughts and feelings more effectively, increase self-esteem and improve the ability to handle anger (33). Another study showed that teens who take part in weekly extracurricular activities, including art, had less anxiety and depression and were generally more optimistic (34). Similarly, participants suggested that physical activities such as running and team sports were beneficial to reduce stress and improve energy and mood. This finding is consistent with the results of a systematic review showing sports are associated with an improvement in psychosocial health (35).

Implications

For peer review only - http://bmjopen.bmj.com/site/about/guidelines.xhtml

The findings may have several implications. For reducing anxiety and depression of an individual or on a group level, not all of the resources listed in this study are likely to be equally available or relevant in a given context However, the eight categories may provide a framework that can help to consider different options for developing programmes that facilitate and strengthen some of the individual and social resources and activities that may help young people to overcome their distress in lower income urban settings.

The findings show that young people come up with a wide range of ideas and experiences about helpful resources. Thus, when considering approaches to improve the mental health of young people, their voice should be heard and take a central role in the development of policies (36). When their views are elicited, innovative methods like arts workshops may help to engage young people and generate helpful material. Based on the experiences in this study, conducting such workshops and interviews online appears a feasible and valuable method to provide a range of findings. Online methods may help to complete similar studies with fewer resources and in shorter periods of time than studies with conventional in-person interviews, thus making them more feasible, particularly in low-resource settings.

Most of the young people in our study reported to have experienced anxiety and/or depression and more than a third had received some type of treatment, although there is no information about the type of treatment. In any case, young people reported largely positive experiences of mental health professionals. Future policies to improve the mental health of young people in large cities should address the need for accessible and effective mental health services. However, this cannot be the sole focus as the provision of mental health services requires substantial funding and using professionals can come with the implicit message that the affected young person cannot cope on their own, which in turn may undermine confidence rather than strengthen it. On a population level, promoting personal and interpersonal skills at school, support from peers and groups, and access to various activities appear more important to reduce the overall level of mental distress in young people.

Finally, considering that this study was carried out in urban environments, it is important to stress the value of community spaces where young people are able to play sports, meet, engage in enjoyable group activities and distract themselves such as parks, public libraries or theatres. In addition to the accessibility of appropriate space, there should also be community organisations enabling and facilitating different activities, especially since some sports and arts activities require equipment and guidance. Future research may explore to what extent community organisations should be set up and supported to attract, engage and help particularly vulnerable and mentally distressed young people.

Conclusion

For peer review only

For peer review only - http://bmjopen.bmj.com/site/about/guidelines.xhtml

Anxiety and depression are frequent among young people. This study shows that in a challenging context like large cities in Latin America, there is a wide range of resources, similar across the three cities, that young people can use and report as helpful. Future research and policies may focus on how these resources can be made widely available, be strengthened and be flexibly utilised by young people if and when required.

Declarations:

Ethics approval and consent to participate:

Written approval was received for this study from the Faculty of Medicine - Research and Ethics Committee of the Pontificia Universidad Javeriana, Bogota on 20/03/2020 (FM-CIE-0241-20) from the Institutional Ethics Committee on Research of the Universidad Peruana Cayetano Heredia, Lima on 31/03/2020 (Constancia E021-03-20), and from the Queen Mary Ethics of Research Committee on 16/11/2020 (QMERC2020/02). All methods were carried out in accordance with relevant guidelines and regulations. All informed consent/assent was obtained from all subjects and/or their legal guardian(s) for participants aged less than 16 years.

Availability of data and materials:

The consent forms did not specify that the data would be deposited in a public repository, and for this reason we are unable to deposit the data. The de-identified participant dataset will be made available from the corresponding author (Natalia Godoy Casasbuenas, natalia.godoy@javeriana.edu.co) upon reasonable request and subject to a data sharing agreement.

Funding:

This work is supported by the Medical Research Council (grant number: MR/S03580X/1)

Authors' contributions:

SP, LIB, FDC, CGR, JMUR and PH devised the project, the main conceptual ideas and design of the study.

MT, NGC, NO, FC, LHP, CGR, JMUR and FDC participated in data collection during the artistic workshops and focus groups.

MT, NGC, LHP, NO, FC participated in the transcription and data analysis of the structured group conversations and focus groups.

MT, NGC, NO wrote the manuscript with input from all authors.

Competing interests: None declared

Acknowledgements:

For peer review only - http://bmjopen.bmj.com/site/about/guidelines.xhtml

We thank Santiago Lucchetti, Jorge Pasquini, Natalia Ortiz, Karen Ariza and Diliniya Stanislaus Sureshkumar for their contributions in the analysis stage. We thank Victoria Jane Bird for comments on the manuscript.

We would like to acknowledge our arts organisations for their contribution to the OLA programme, Crear Vale La Pena, Fundación Batuta, Familia Ayara, Arenas y Esteras and Teatro La Plaza.

References

1. Somerville LH, Jones RM, Casey BJ. A time of change: behavioral and neural correlates of adolescent sensitivity to appetitive and aversive environmental cues. Brain Cogn. 2010 Feb;72(1):124–33.

2. Spear LP. The adolescent brain and age-related behavioral manifestations. Neurosci Biobehav Rev. 2000 Jun;24(4):417–63.

3. Lopez V, Sanchez K, Killian MO, Eghaneyan BH. Depression screening and education: an examination of mental health literacy and stigma in a sample of Hispanic women [Internet]. Vol. 18, BMC Public Health. 2018. Available from: http://dx.doi.org/10.1186/s12889-018-5516-4

4. van der Heide I, Wang J, Droomers M, Spreeuwenberg P, Rademakers J, Uiters E. The relationship between health, education, and health literacy: results from the Dutch Adult Literacy and Life Skills Survey. J Health Commun. 2013;18 Suppl 1:172–84.

5. Benjet C, Sampson L, Yu S, Kessler RC, Zaslavsky A, Evans-Lacko S, et al. Associations between neighborhood-level violence and individual mental disorders: Results from the World Mental Health surveys in five Latin American cities. Psychiatry Res. 2019 Dec;282:112607.

6. Morgan C, Reininghaus U, Reichenberg A, Frissa S, SELCoH study team, Hotopf M, et al. Adversity, cannabis use and psychotic experiences: evidence of cumulative and synergistic effects. Br J Psychiatry. 2014 Mar 13;204:346–53.

7. World Health Organization. Adolescent mental health. Fact sheet [internet]. 2021 [updated 2021 November 17; cited 2021 November 30]. Available from: https://www.who.int/news-room/fact-sheets/detail/adolescent-mental-health

8. Adwas A, Jbireal J, Azab A. Anxiety: Insights into Signs, Symptoms, Etiology, Pathophysiology, and Treatment. The South African journal of medical sciences. 2019;2:80-91.

9. Caqueo-Urízar A, Flores J, Escobar C, Urzúa A, Irarrázaval M. Psychiatric disorders in children and adolescents in a middle-income Latin American country [Internet]. Available from: http://dx.doi.org/10.21203/rs.2.14739/v2

10. Van Ameringen M, Mancini C, Farvolden P. The impact of anxiety disorders on educational

For peer review only - http://bmjopen.bmj.com/site/about/guidelines.xhtml

achievement. Journal of anxiety disorders. 2003 Jan 1;17(5):561-71.

11. Patel V, Flisher AJ, Hetrick S, McGorry P. Mental health of young people: a global public-health challenge. The Lancet. 2007 Apr 14;369(9569):1302-13.

12. Kim D. Blues from the neighborhood? Neighborhood characteristics and depression. Epidemiologic reviews. 2008 Nov 1;30(1):101-17.

13. Morgan C, Reininghaus U, Reichenberg A, Frissa S, Hotopf M, Hatch SL, SELCoH Study Team. Adversity, cannabis use and psychotic experiences: evidence of cumulative and synergistic effects. The British Journal of Psychiatry. 2014 May;204(5):346-53.

14. Thapar A, Collishaw S, Pine DS, Thapar AK. Depression in adolescence. The Lancet. 2012 Mar 17;379(9820):1056-67.

15. Murcia EH, Losada YL, Rojas LA, Londoño PG. Prevalencia de la depresión infantil en Neiva (Colombia). Avances en Psicología Latinoamericana. 2009 Sep 23;27(1):154-64.

16. Méndez X, Rosa AJ, Montoya M, Espada JP, Olivares J, Sánchez-Meca J. Tratamiento Psicológico de la Depresión Infantil. Psicología conductual. 2002;10(3):563-80.

17. March J, Silva S, Petrycki S, Curry J, Wells K, Fairbank J, et al. Fluoxetine, cognitive-behavioral therapy, and their combination for adolescents with depression: Treatment for Adolescents With Depression Study (TADS) randomized controlled trial. JAMA [Internet]. 2004 Aug 18 [cited 2021 Jul 12];292(7). Available from: https://pubmed.ncbi.nlm.nih.gov/15315995/

18. Stapinski LA, Montgomery AA, Heron J, Jerrim J, Vignoles A, Araya R. Depression symptom trajectories and associated risk factors among adolescents in Chile. PLoS One. 2013 Oct 11;8(10):e78323.

19. Anderson K, Priebe S. Concepts of resilience in adolescent mental health research. Journal of Adolescent Health. 2021 May 24.

20. Ayed N, Toner S, Priebe S. Conceptualizing resilience in adult mental health literature: A systematic review and narrative synthesis. Psychol Psychother. 2019 Sep;92(3):299–341.

21. Zarobe L, Bungay H. The role of arts activities in developing resilience and mental wellbeing in children and young people a rapid review of the literature [Internet]. Vol. 137, Perspectives in Public Health. 2017. p. 337–47. Available from: http://dx.doi.org/10.1177/1757913917712283

22. Hildebrand NA, Celeri EHRV, Morcillo AM, Zanolli M de L. Resilience and mental health problems in children and adolescents who have been victims of violence. Rev Saude Publica. 2019 Jan 31;53:17.

23. Suarez EB. Two decades later: The resilience and post-traumatic responses of Indigenous Quechua girls and adolescents in the aftermath of the Peruvian armed conflict. Child Abuse Negl. 2013 Feb;37(2-3):200–10.

For peer review only - http://bmjopen.bmj.com/site/about/guidelines.xhtml

24. Vera-Bachmann D. [Resilience, Poverty and Rurality]. Rev Med Chil. 2015 May;143(5):677–8.

25. Herrera-Moreno D, Carvajal-Ovalle D, Cueva-Nuñez MA, Acevedo C, Riveros-Munévar F, Camacho K, et al. Body image, perceived stress, and resilience in military amputees of the internal armed conflict in Colombia. Int J Psychol Res (Medellin). 2018 Jul;11(2):56–62.

26. Miller-Graff LE, Scheid CR, Guzmán DB, Grein K. Caregiver and family factors promoting child resilience in at-risk families living in Lima, Peru. Child Abuse Negl. 2020 Oct;108:104639.

27. Creswell JW, Hanson WE, Clark Plano VL, Morales A. Qualitative research designs: Selection and implementation. The counseling psychologist. 2007; 35(2):236-264.

28. Priebe S, Fung C, Brusco LI, Carbonetti F, Gómez-Restrepo C, Uribe M, Diez-Canseco F, Smuk M, Holt N, Kirkbride JB, Araya R. Which resources help young people to prevent and overcome mental distress in deprived urban areas in Latin America? A protocol for a prospective cohort study. BMJ open. 2021 Sep 1;11(9):e052339.

29. Bengtsson M. How to plan and perform a qualitative study using content analysis. NursingPlus Open. 2016 Jan 1;2:8-14.

30. Gaete J, Labbé N, Del Villar P, Allende C, Araya R, Valenzuela E. Mental health and associated factors among young offenders in Chile: a cross-sectional study. Crim Behav Ment Health. 2018 Feb;28(1):13–27.

31. Kuznesof EA. The House, the Street, Global Society: Latin American Families and Childhood in the Twenty-First Century [Internet]. Vol. 38, Journal of Social History. 2005. p. 859–72. Available from: http://dx.doi.org/10.1353/jsh.2005.0065

32. Parra A, Oliva A, del Carmen Reina M. Family Relationships From Adolescence to Emerging Adulthood [Internet]. Vol. 36, Journal of Family Issues. 2015. p. 2002–20. Available from: http://dx.doi.org/10.1177/0192513x13507570

33. Uttley L, Scope A, Stevenson M, Rawdin A, Taylor Buck E, Sutton A, et al. Systematic review and economic modelling of the clinical effectiveness and cost-effectiveness of art therapy among people with non-psychotic mental health disorders. Health Technol Assess. 2015 Mar;19(18):1–120, v – vi.

34. Thomson LJ, Morse N, Elsden E, Chatterjee HJ. Art, nature and mental health: assessing the biopsychosocial effects of a “creative green prescription” museum programme involving horticulture, artmaking and collections. Perspect Public Health. 2020 Sep;140(5):277–85.

35. Eime RM, Young JA, Harvey JT, Charity MJ, Payne WR. A systematic review of the psychological and social benefits of participation in sport for children and adolescents: informing development of a conceptual model of health through sport. Int J Behav Nutr Phys Act. 2013 Aug 15;10:98.

36. Oxman AD, Lewin S, Lavis JN, Fretheim A. SUPPORT Tools for evidence-informed health

For peer review only - http://bmjopen.bmj.com/site/about/guidelines.xhtml

Policymaking (STP) 15: Engaging the public in evidence-informed policymaking. Health Res Policy Syst. 2009 Dec 16;7 Suppl 1:S15.

For peer review only - http://bmjopen.bmj.com/site/about/guidelines.xhtml

OLA WP1 Arts-based workshops: Topic guide for structured conversations with adolescents and young adults

Objectives: (1) To explore the experiences of emotional and mental health problems, including feelings of depression, anxiety, and stress of young people with emphasis on understanding how these episodes have been recovered and overcome. (2) Explore what role they think arts and creativity had or could have had to protect them and help them recover from anxiety and depression.

a. Breaking the ice

What caught your attention in the theatre?
If theatre is removed from your life, what would you lose?

b. Recognizing mental anguish

We want to know what emotional and mental health problems (anxiety / depression / stress) mean for you.

Have you ever experienced it? If not, have you perceived it in people close to you?

How do you experience it? Can you visualize / express it? Can you communicate it to others? Have you found metaphors or symbols or arts to express these feelings?

Have you found any artistic resource to express these feelings, such as workshops, poetry, music, etc?

c. Coping with mental anguish

Where do you go when you feel distressed? Real places? Fantasy worlds? Are you looking for help in your environment? Do you attend reflection groups? Individual therapy? Group therapy? Do you go to Church? Do you play sport or take physical exercise? Or do you take refuge in yourselves? Do you use your imagination? Do you make art?

How do you physically experience anxiety / distress? What other impact does it have on your life?

Who do they talk to about these feelings? Other young people? Friends? Teachers? Families? Where? When? Who can help best in these moments?

Can you map out the resources (people / places / activities) in your daily life that allow you to reduce distressed state and acquire greater strength in triggering situations? What keeps you safe and comfortable? What significant physical and mental changes do you think you have experienced? How could them describe this mood change?

For peer review only

The recovery process is not always linear or progressive, it involves progress and Setbacks. Can you identify what are the situations or elements that influence those setbacks?

What emotions or feelings do you perceive when moving from anxiety to emotional stability?

d. Understanding the relationship between art and resilience

How do the arts and creative practices build your ability to recover from anxiety and distress as well as to develop resilience to mental pathology?

For peer review only

OLA WP1: Topic guide for focus groups with adolescents, young adults and professionals to explore views on resources that young people use to overcome mental distress

Introduction and group rules (5 mins)

Thank participants for their availability, introduce researchers and explain:

- All participants here have signed a consent form that describes the study and the aim of the group.
- Aim of the group: to explore and understand your views on the types of resources that young people use to overcome mental and emotional health problems. By resources we mean what helps people to recover, specifically personal resources (for example, what people do and how people might cope in different situations), and social resources (for example, your friendships).
- We are interested in seeing if the resources that young people use to recover from mental and emotional health problems change as they grow older; and so we are involving young people in our study who are aged 15-16 and 20-24 years.
- Confidentiality: the names of the participants will only be known by the researchers and other participants in today's focus group, and will not be revealed to anyone else.
- The session will be audio-recorded, transcribed and analysed by researchers.
- All participants will be assigned an ID number and all potentially identifying information will be removed.
- Audio recordings will be destroyed once data analysis is complete.
- One researcher will facilitate the discussion using a topic guide. The second researcher will make sure all areas to be discussed are covered and to write notes about important points raised.

<u>Outline group rules</u>:

- Information shared within the group must remain confidential. We ask that you try not to use names if talking about services or staff members.
- Speaking one at a time and listening and respecting others' opinions
- You do not have to share information that you do not want to
- There are no right or wrong answers
- We are looking for the widest possible range of views. Alignment of views within the group is not required
- Mobile phones to be switched off or on silent mode (unless required for work/urgent personal reasons)

- Any questions?
- Start audio recorder

Ice breaker (approx. 5 mins):

Ask each participant in turn to say:

For peer review only

- Their name or nickname
- And something about themselves, e.g. one thing they enjoy doing or a talent they have

Focus group discussion (approx. 45 mins):
Aim: to explore and understand the views and experiences of adolescents, young adults and professionals on the types of resources that young people use, alternative ways of measuring resources and on the resources included in the assessment battery.

During adolescence and youth, it can be common for people to experience mental and emotional health problems, which includes feelings of depression, anxiety and stress. Living in big cities can mean people are more likely to experience stressful events. However, not all people develop mental and emotional health problems, and when people do become emotionally distressed, many will recover within a year. And so we want to understand what helps people to recover.

<u>Introduction:</u>
- <u>What are the situations or experiences that might cause emotional distress in young people?</u>

<u>Types of resources</u>:
- What helps young people to recover from mental and emotional health problems?
- What resources do young people have in their daily life that help with feelings of emotional distress?
- What helps people if they are feeling down, upset or anxious?

Prompts:
Family/partner
Friendships
Groups in community such as religious groups, youth centres
Sports activities – alone or in groups
Arts activities – alone or in groups
Internet/social media
Personal resources (thinking styles, coping strategies, self-care)
Green spaces in cities/going out of the city/contact with nature
Can you think of other things that help people?

- How do they help?
  Prompts:
  Do they help all people, or just some people?
  - Are there resources that are more helpful for young people depending on their gender?
  - What resources are more helpful for young people of male/female/non binary gender?
  - Are there resources that are more helpful for your age group compared to the younger/older age group?

- Are there resources in the local community for young people?

For peer review only

   - What type of resources

- How do young people learn about and access resources in the local community?
   - How do people find out about these resources?
   - Do they need a referral to start attending a group, club or organisation?
   - Do they need to pay a fee?

- What are your/young people's experiences of accessing these resources?
  Prompts:
  Was it easy to access them and why?
  Did you face any barriers or difficulties?
- What resources would young people like to see and access in the local community?

<u>Measuring resources</u>:
In this research study, we would like to understand better the resources that help young people to overcome mental and emotional health problems. To help with this, we have put together a booklet to ask young people about the resources that they use. The booklet consists of a range of questionnaires that can be completed by young people. At the moment, the resources that are included in the booklet are: (show and read through list of resources).

- In your opinions, are there resources or areas that are important for mental and emotional health to young people, which are missing from the booklet?
   - What resources?
   - Why?
   - How would you ask young people about these resources?

- Are there resources in the booklet, which you think are not relevant for young people?
   - What resources?
   - Why?

- Do you have any other thoughts or comments about the resources in the booklet?

- We currently plan to ask young people about these resources using a booklet of questionnaires. What do you think about this approach?
   - How should young people complete this booklet of questionnaires? (by themselves, with the help of a researcher?)

- What other ways do you think we could use to identify and ask about the resources that young people use, apart from this booklet?
   - Can you think of other approaches to explore resources with young people?

Conclusion (up to 10 mins)

For peer review only

- Does anyone have anything else to add to what has been discussed today: any further comments or recommendations about resources for recovering from mental and emotional health problems?
- Does anyone have questions for us about this study?
- Thank participants for their contribution

For peer review only

Reflexivity Statement

1. How does this study address local research and policy priorities?

The study explores which personal and social resources young people for deprived neighbourhoods in large Latin American cities use to overcome episodes of anxiety and depression. Growing up in deprived neighbourhoods is associated with a number of risk factors for developing mental disorders such as poverty, violence and social inequality. Anxiety and depression are frequent among young people in those neighbourhoods, but evidence suggests that about 50% of affected people overcome their distress within a year. The study explored which resources they use in that process. The mental health of young people is a societal priority across the world, and the challenge of improving it has become even more difficult during the pandemic. Since not all young people can or should access professional health services for help, a good understanding of what resources they use and find helpful is essential. Thus, the study addresses an area of utmost relevance for local policies and health.

2. How were local researchers involved in study design?

The study was developed in a group process with researchers from Bogotá, Buenos Aires and Lima as equal partners. Most decisions were taken in a meeting in Lima in 2019 where consensus was reached on the study design. Since the study was conducted in Bogotá, Buenos Aires and Lima, the views of the local researchers were central to the design of the study and to its organisation, and they also led on collaborating with local arts organisations.

3. How has funding been used to support the local research team?

Most of the funding went to the local research teams and their activities on the study were fully funded by the grant. How precisely they used the funding in terms of contracts with research assistants and support for activities was the decision of local teams and varied across the participating cities.

4. How are research staff who conducted data collection acknowledged?

There are three co-authors from each partner, who led on the data collection. In the acknowledgement we also mention all the research assistants who were involved in the data collection although their role did not meet the criteria for authorship.

5. Do all members of the research partnership have access to study data?

Yes.

6. How was data used to develop analytical skills within the partnership?

The data was analysed by the local research teams. Since the material is in Spanish, only they were able to analyse all the data in full. They conducted the analysis and drafted the first version of the paper, involving researchers from all three partner cities. Subsequently, the leading authors are from the partners in Latin America.

7. How have research partners collaborated in interpreting study data?

For peer review only

They led on the interpretation of the data and wrote the first version of the paper, with the team in London providing support.

8. How were research partners supported to develop writing skills?

The paper was written by the research partners and then discussed, further developed and improved in an iterative communication with the Chief Investigator from London. Thus, the researchers leading on the paper in the partner countries received on-going feed-back and were involved in all steps of the editing of the paper.

9. How will research products be shared to address local needs?

We believe that some of the results are of policy relevance to improve the mental health of young people in deprived areas in Latin American cities. Once published, we will disseminate the findings further using social media, personal communications, presentations and links of the partners with local policy makers.

10. How is the leadership, contribution and ownership of this work by LMIC researchers recognised within the authorship?

There are 12 authors in total. First authorship is shared between the leading author from Colombia and Peru with the leading research from Argentina being the third author. There are two further researchers from each partner as authors (in alphabetical order). The only authors not from the partners are the co-ordinator of the participating arts organisations across Latin America (PH), the programme manager supporting all activities and co-ordinating communication across sites throughout the study (CF), and the Chief Investigator of the study who provided guidance and support throughout the process, including the paper writing stage.

11. **How have early career researchers across the partnership been included within the authorship team?**

There are five early career researchers among the authors, including the three leading authors (MT, NGC, NO).

12. How has gender balance been addressed within the authorship?

First authorship is shared between a female and a male researcher. The corresponding author is female. The third author leading for Argentina is female. Overall, there are eight men and four women among the authors.

13. How has the project contributed to training of LMIC researchers?

The study is part of a research programme with a strong emphasis on the training and promotion of early career researchers in Latin America. The funding provides them with the possibility to focus on research and develop their skills in research. Various components of the programme contributed that which includes specific seminars on critical appraisal and paper writing.

14. How has the project contributed to improvements in local infrastructure?

The significant funding of the research programme has established an infrastructure for mental health research that had not existed in the same way before. The exact context and set up vary

For peer review only

across partners. Whilst all partners aim to make the infrastructure sustainable, it remains to be seen how much of the new infrastructure can be maintained beyond the end of the research programme.

15. What safeguarding procedures were used to protect local study participants and researchers?

The safeguarding procedures met international standards and were specified in the applications to the ethics committee in each country and in London.

For peer review only

Standards for Reporting Qualitative Research (SRQR)*

http://www.equator-network.org/reporting-guidelines/srqr/

Title and abstract

	Page/line no(s).
**Title** - Concise description of the nature and topic of the study Identifying the study as qualitative or indicating the approach (e.g., ethnography, grounded theory) or data collection methods (e.g., interview, focus group) is recommended	Page 0 – Line 1
**Abstract** - Summary of key elements of the study using the abstract format of the intended publication; typically includes background, purpose, methods, results, and conclusions	Page 1

Introduction

**Problem formulation** - Description and significance of the problem/phenomenon studied; review of relevant theory and empirical work; problem statement	Page 3
**Purpose or research question** - Purpose of the study and specific objectives or questions	Page 3 – Lines 30-32

Methods

**Qualitative approach and research paradigm** - Qualitative approach (e.g., ethnography, grounded theory, case study, phenomenology, narrative research) and guiding theory if appropriate; identifying the research paradigm (e.g., postpositivist, constructivist/ interpretivist) is also recommended; rationale**	Page 3 – Line 35
**Researcher characteristics and reflexivity** - Researchers' characteristics that may influence the research, including personal attributes, qualifications/experience, relationship with participants, assumptions, and/or presuppositions; potential or actual interaction between researchers' characteristics and the research questions, approach, methods, results, and/or transferability	Page 5 – Lines 22-24
**Context** - Setting/site and salient contextual factors; rationale**	Page 4 – Lines 26-29
**Sampling strategy** - How and why research participants, documents, or events were selected; criteria for deciding when no further sampling was necessary (e.g., sampling saturation); rationale**	Page 4 – Lines 26-29
**Ethical issues pertaining to human subjects** - Documentation of approval by an appropriate ethics review board and participant consent, or explanation for lack thereof; other confidentiality and data security issues	Page 6 – Lines 17-21
**Data collection methods** - Types of data collected; details of data collection procedures including (as appropriate) start and stop dates of data collection and analysis, iterative process, triangulation of sources/methods, and modification of procedures in response to evolving study findings; rationale**	Page 4 – Lines 11-25<br>Page 5 – Lines 14-34<br>Page 6 – Lines 1-8

For peer review only - http://bmjopen.bmj.com/site/about/guidelines.xhtml

**Data collection instruments and technologies** - Description of instruments (e.g., interview guides, questionnaires) and devices (e.g., audio recorders) used for data collection; if/how the instrument(s) changed over the course of the study	Page 5 – Lines 6-13
**Units of study** - Number and relevant characteristics of participants, documents, or events included in the study; level of participation (could be reported in results)	Page 6 – Lines 23-31
**Data processing** - Methods for processing data prior to and during analysis, including transcription, data entry, data management and security, verification of data integrity, data coding, and anonymization/de-identification of excerpts	Page 5 – Lines 26-27
**Data analysis** - Process by which inferences, themes, etc., were identified and developed, including the researchers involved in data analysis; usually references a specific paradigm or approach; rationale**	Page 5 – Lines 25-34<br>Page 6 – Lines 1-8
**Techniques to enhance trustworthiness** - Techniques to enhance trustworthiness and credibility of data analysis (e.g., member checking, audit trail, triangulation); rationale**	Page 5 – Lines 29-34<br>Page 6 – Lines 1-4

Results/findings

**Synthesis and interpretation** - Main findings (e.g., interpretations, inferences, and themes); might include development of a theory or model, or integration with prior research or theory	Pages 8-12
**Links to empirical data** - Evidence (e.g., quotes, field notes, text excerpts, photographs) to substantiate analytic findings	Pages 8-12

Discussion

**Integration with prior work, implications, transferability, and contribution(s) to the field -** Short summary of main findings; explanation of how findings and conclusions connect to, support, elaborate on, or challenge conclusions of earlier scholarship; discussion of scope of application/generalizability; identification of unique contribution(s) to scholarship in a discipline or field	Pages 12-15
**Limitations** - Trustworthiness and limitations of findings	Pages 13-14

Other

**Conflicts of interest** - Potential sources of influence or perceived influence on study conduct and conclusions; how these were managed	None declared Page 17 Line 37
**Funding** - Sources of funding and other support; role of funders in data collection, interpretation, and reporting	Page 16 – Lines 21-22

*The authors created the SRQR by searching the literature to identify guidelines, reporting standards, and critical appraisal criteria for qualitative research; reviewing the reference lists of retrieved sources; and contacting experts to gain feedback. The SRQR aims to improve the transparency of all aspects of qualitative research by providing clear standards for reporting qualitative research.

For peer review only - http://bmjopen.bmj.com/site/about/guidelines.xhtml

**The rationale should briefly discuss the justification for choosing that theory, approach, method, or technique rather than other options available, the assumptions and limitations implicit in those choices, and how those choices influence study conclusions and transferability. As appropriate, the rationale for several items might be discussed together.

Reference:

O'Brien BC, Harris IB, Beckman TJ, Reed DA, Cook DA. **Standards for reporting qualitative research: a synthesis of recommendations.** *Academic Medicine*, Vol. 89, No. 9 / Sept 2014 DOI: 10.1097/ACM.0000000000000388

For peer review only

BMJ Open

Identifying resources used by young people to overcome mental distress in three Latin American cities: A qualitative study

Journal:	*BMJ Open*
Manuscript ID	bmjopen-2021-060340.R2
Article Type:	Original research
Date Submitted by the Author:	06-Jul-2022
Complete List of Authors:	Toyama, Mauricio; Universidad Peruana Cayetano Heredia, CRONICAS Center of Excellence in Chronic Diseases<br>Godoy-Casasbuenas, Natalia; Pontificia Universidad Javeriana, Department of Clinical Epidemiology and Biostatistics<br>Olivar, Natividad; Universidad de Buenos Aires, Department of Psychiatry and Mental Health, School of Medicine<br>Brusco, Luis Ignacio; Universidad de Buenos Aires, Department of Psychiatry and Mental Health, School of Medicine<br>Carbonetti, Fernando; Universidad de Buenos Aires, Department of Psychiatry and Mental Health, School of Medicine<br>Diez-Canseco, Francisco; Universidad Peruana Cayetano Heredia, CRONICAS Center of Excellence in Chronic Diseases<br>Gómez-Restrepo, Carlos; Pontificia Universidad Javeriana, Department of Clinical Epidemiology and Biostatistics; Pontificia Universidad Javeriana, Department of Psychiatry and Mental Health<br>Heritage, Paul; Queen Mary University of London School of English and Drama<br>Hidalgo-Padilla, Liliana; Universidad Peruana Cayetano Heredia, CRONICAS Center of Excellence in Chronic Diseases<br>Uribe, Miguel ; Pontificia Universidad Javeriana, Department of Psychiatry and Mental Health<br>Steffen, Mariana ; Queen Mary University of London, People's Palace Projects. c/o School of English and Drama, QMUL Mile End Road, London E1 4NS<br>Fung, Catherine ; Queen Mary University of London, Unit for Social and Community Psychiatry<br>Priebe, Stefan; Queen Mary University of London, Unit for Social and Community Psychiatry
**Primary Subject Heading**:	Mental health
Secondary Subject Heading:	Qualitative research, Public health, Global health
Keywords:	MENTAL HEALTH, QUALITATIVE RESEARCH, Child & adolescent psychiatry < PSYCHIATRY, Depression & mood disorders < PSYCHIATRY

For peer review only - http://bmjopen.bmj.com/site/about/guidelines.xhtml

I, the Submitting Author has the right to grant and does grant on behalf of all authors of the Work (as defined in the below author licence), an exclusive licence and/or a non-exclusive licence for contributions from authors who are: i) UK Crown employees; ii) where BMJ has agreed a CC-BY licence shall apply, and/or iii) in accordance with the terms applicable for US Federal Government officers or employees acting as part of their official duties; on a worldwide, perpetual, irrevocable, royalty-free basis to BMJ Publishing Group Ltd ("BMJ") its licensees and where the relevant Journal is co-owned by BMJ to the co-owners of the Journal, to publish the Work in this journal and any other BMJ products and to exploit all rights, as set out in our licence.

The Submitting Author accepts and understands that any supply made under these terms is made by BMJ to the Submitting Author unless you are acting as an employee on behalf of your employer or a postgraduate student of an affiliated institution which is paying any applicable article publishing charge ("APC") for Open Access articles. Where the Submitting Author wishes to make the Work available on an Open Access basis (and intends to pay the relevant APC), the terms of reuse of such Open Access shall be governed by a Creative Commons licence – details of these licences and which Creative Commons licence will apply to this Work are set out in our licence referred to above.

Other than as permitted in any relevant BMJ Author's Self Archiving Policies, I confirm this Work has not been accepted for publication elsewhere, is not being considered for publication elsewhere and does not duplicate material already published. I confirm all authors consent to publication of this Work and authorise the granting of this licence.

For peer review only

Title: Identifying resources used by young people to overcome mental distress in three Latin American cities: A qualitative study

Corresponding author:
Natalia Godoy-Casasbuenas
Postal address: Carrera 7ª # 40-62, 2nd floor – Bogotá, Colombia
E-mail: natalia.godoy@javeriana.edu.co
Telephone: (+571) 3208320 ext. 2758

Authors:
Mauricio Toyama[1], Natalia Godoy-Casasbuenas[2], Natividad Olivar[3], Luis Ignacio Brusco[3], Fernando Carbonetti[3], Francisco Diez-Canseco[1], Carlos Gómez-Restrepo[2,4], Paul Heritage[5], Liliana Hidalgo-Padilla[1], José Miguel Uribe-Restrepo[4], Mariana Steffen[6], Catherine Fung[7], Stefan Priebe[7]

Authors information:
[1]CRONICAS Centre of Excellence in Chronic Diseases, Universidad Peruana Cayetano Heredia, Lima, Perú

[2]Department of Clinical Epidemiology and Biostatistics, Pontificia Universidad Javeriana, Bogotá, Colombia

[3]Department of Psychiatry and Mental Health, School of Medicine, Universidad de Buenos Aires, Buenos Aires, Argentina

[4]Department of Psychiatry and Mental Health, Pontificia Universidad Javeriana, Bogotá, Colombia

[5]School of English and Drama, Queen Mary University of London, London, United Kingdom

[6]Queen Mary University of London, People's Palace Projects. c/o School of English and Drama, QMUL Mile End Road, London E1 4NS

[7]Unit for Social and Community Psychiatry (WHO Collaborating Centre for Mental Health Service Development), Queen Mary University of London, London, United Kingdom

Key words: Mental Health, Adolescent psychiatry, Depression, Anxiety, Qualitative Research

Abstract count: 295
Word count: 5312

Number of references: 36

Abstract (295 words):

Objective: To explore which resources and activities help young people living in deprived urban environments in Latin America to recover from depression and/or anxiety.

Design: Multimethod, qualitative study with 18 online focus groups and 12 online structured group conversations embedded into arts workshops.

Setting: This study was conducted in Bogotá (Colombia), Buenos Aires (Argentina) and Lima (Peru)

Participants: Adolescents (15-16 years old) and young adults (20-24 years old) with capacity to provide assent/consent; and professionals (older than 18 years of age) that had experience of professionally working with young people, were willing to share personal experience within a group, and had capacity to provide consent.

Results: A total of 185 participants took part in this study: 111 participants (36 adolescents, 35 young adults and 40 professionals) attended the 18 focus groups; and 74 young people (29 adolescents and 45 young adults) took part in the 12 arts workshops. Eight categories captured the resources and activities that were reported by young people as helpful to overcome mental distress: i) Personal resources, ii) Personal development, iii) Spirituality and religion, iv) Social resources, v) Social media, vi) Community resources, vii) Activities (sub-categorized into artistic, leisure, sports and outdoor activities) and viii) Mental health professionals. Personal and social resources as well as artistic activities and sports were the most common resources identified that help adolescents and young adults to overcome depression and anxiety.

Conclusion: Despite the different contexts of the three cities, young people appear to use similar resources to overcome mental distress. Policies to improve the mental health of young people in deprived urban settings should address the need of community spaces where young people can play sports, meet, and engage in groups, and support community organisations that can enable and facilitate a range of social activities.

Strengths and limitations

- This study explored which resources young people use to overcome mental distress in three large urban Latin American cities employing Spanish as the same language which facilitated the analysis across countries and allowed for direct comparisons.

- The study combined different perspectives (adolescents, young adults, professionals) and methods (focus groups, structured group conversations embedded in arts workshops) providing a different approach to engage young people and encourage them to express their views.

For peer review only - http://bmjopen.bmj.com/site/about/guidelines.xhtml

- The recruitment of a substantial proportion of participants was done through arts organisations which may have biased the findings, leading to a particularly strong emphasis on arts activities.
- The study was conducted during the Covid-19 pandemic which may have influenced the views of young people as to what they find helpful.
- Focus groups and structured group conversations were conducted online, which may have influenced the interaction between the facilitators and the participants and thus the results.

DRAFT
For peer review only

Introduction

Adolescence and young adulthood represent a period of growth and development. Young people experience physical changes as well as changes in their cognitions, emotions and social environment (1,2). At the same time, cultural, socioeconomic, and environmental influences, including poverty, low education (3,4), violence (5), or substance use (6), make adolescents and young adults particularly vulnerable to developing mental health problems.

According to the World Health Organization (WHO), it is estimated that between 10% and 20% of young people experience mental disorders, most commonly depression and anxiety disorders (7). Depression represents a common of illness and disability in this group, can be associated with substance abuse, aggressiveness, eating disorders, and is one of the main risk factors for suicide. Anxiety can be accompanied by disturbances of sleep, concentration, social and/or occupational functioning (8), and is the ninth cause of illness and disability in this population (9). Poor mental health at young age is associated with further health and social problems such as poorer academic achievement, early school drop-out and substance abuse (10).

Young people living in major cities across Latin America can be exposed to various risk factors for developing mental disorders, such as poverty, violence and social inequality (11-14). The percentage of young people with depression and/or anxiety has been reported to range from 17% in Colombia (15) to 26% in Argentina (16). However, longitudinal studies have found that among young people who suffer from depression and anxiety, between 50 and 60% manage to overcome their distress within one year (17,18).

The ability to overcome mental distress – sometimes referred to as resilience - is linked to personal resources such as motivation and hope and to social resources such as support from peers and access to activities in the surrounding environment (19, 20). In children and young adults, arts activities can be an important social resource (21). In Latin America, studies about resources to cope with adversity have been carried out in the context of social violence (22,23), poverty (24), forced displacement (25) and medical conditions (26), but to our knowledge no study has explored the resources that help young people in this region to overcome mental distress.

The aim of this study is to explore the participant´s perceptions of which resources and activities help young people in large cities in Latin America to recover from mental distress in form of depression and/or anxiety, using both conventional focus groups and arts workshops.

Methods

Study design

This is an exploratory qualitative study using a grounded theory design (27). The study was conducted within the context of a larger research programme called "Building resilience and

For peer review only - http://bmjopen.bmj.com/site/about/guidelines.xhtml

resources to reduce depression and anxiety in young people from urban neighbourhoods in Latin America (OLA)". The overall aim of the programme is to identify which characteristics, resources and activities help young people living in urban environments in Latin America to prevent or recover from depression and/or anxiety (28). The programme is a collaboration between Queen Mary, University of London (UK), Universidad de Buenos Aires (Argentina), Pontificia Universidad Javeriana in Bogotá (Colombia) and Universidad Peruana Cayetano Heredia in Lima (Peru). The researchers work in close partnerships with the following arts organisations: Crear Vale La Pena (Argentina), Fundación Batuta and Familia Ayara (Colombia) and Teatro La Plaza (Peru). These organisations specialize in building creative environments, using classical music, rap music, street art, dance, theatre and visual arts, respectively.

This study used two forms of group activities for data collection: focus groups including adolescents (15/16 years), young adults (20-24 years) and professionals working with young people; and arts workshops with young people (15-24 years). The arts workshops were intended as a different approach to engage young people and elicit their views. Towards the end or following the arts activities, all arts workshops included a structured group conversation to explore resources to overcome anxiety and/or depression. Because of the restrictions due to the pandemic, all groups were conducted online between August and November of 2020.

A total of 18 focus groups were conducted: six in each country, two for each group of participants (adolescents, young adults and professionals). The focus groups lasted between approximately 90 and 120 minutes. There were also 12 arts workshops: four in Argentina using a combination of creative activities run by the arts organisation 'Crear Vale La Pena'; two in Colombia using rap and graffiti run by 'Familia Ayara' and four with musical activities run by 'Fundación Batuta'; two in Peru using theatre and dramaturgical exercises run by 'Teatro La Plaza'. The length of the workshops varied. They usually lasted between two and three hours. Each focus group and arts workshop had a facilitator and a co-facilitator or technical supporter.

Study setting

The study was conducted in the capital cities of three Latin American countries - Buenos Aires (Argentina), Bogotá (Colombia) and Lima (Peru) – and focused on young people from poorer economic neighbourhoods based on national indicators of income.

Participants

Three groups of participants were included in the study: adolescents, young adults, and professionals with experience in working with young people.

For the focus groups, the inclusion criteria were: 15-16 years or 20-24 years of age, respectively; willingness to share personal experiences within the group; capacity to provide assent/consent;

For peer review only - http://bmjopen.bmj.com/site/about/guidelines.xhtml

being currently experiencing depression/anxiety defined by having a clinical diagnosis or currently using mental health services and, for the adolescents, consent from a parent/legal guardian

Professionals were included if they were at least 18 years of age, had experience of professionally working with young people, were willing to share personal experience within a group, and had capacity to provide consent.

Inclusion criteria for the arts workshops were: 15-24 years of age, current or past participation in activities of the collaborating arts organisations, capacity to provide assent/consent, and, for those under 18 years old, consent from a parent/legal guardian.

Data collection tools

Basic sociodemographic data and previous experience of anxiety and/or depression were collected from all participants in the focus groups on a brief questionnaire, completed by phone or through videoconference (e.g., Zoom).

Topic guides for both focus groups and structured group conversations within the arts workshopsaddressed the resources used by young people when experiencing mental distress. We introduced the concept of distress using anxiety and depression as examples since they are the most common forms of mental disorders.(See supplementary files 1 and 2). The same topic guides were applied in the three countries.

Procedures

Participants were identified through schools, health services, non-governmental organisations, and youth organisations as well as the four participating arts organisations. Potentially interested participants were contacted via phone by the research team to check the inclusion criteria before they were invited to formally participate in the study. The informed consent procedures were conducted through phone or videoconference. Participants were given the options to provide their consent by phone recording, completing the consent form virtually or completing the form on paper and sending a scan or picture to the research team. Adolescents provided their informed assent, and a parent or legal guardian was contacted to receive their permission and consent. The focus groups and structured group conversations were conducted by researchers trained in qualitative data collection and analysis.

Data analysis

Audio recordings of the focus groups and structured group conversations were transcribed verbatim. We conducted an inductive content analysis using the four stages proposed by Bengtsson (29). The research team from each country analysed the transcriptions of their local participants. At least two members of the research teams in each country worked together during the analysis. During the first stage, decontextualization, the research teams familiarised themselves with the

For peer review only - http://bmjopen.bmj.com/site/about/guidelines.xhtml

For peer review only DRAFT

data and inductively created codes based on the relevant information they identified. These codes were added to a spreadsheet shared between the teams. During the recontextualization stage, the codes and categories were discussed in weekly meetings to standardize the codes across the three countries to ensure reliability. After the meetings, each team revisited their transcriptions to either re-code their coding to match those identified in other countries, or to code new information they may have missed, based on codes created by the other teams. Furthermore, during the categorisation stage, the codes were discussed and grouped into categories based on similarity of content. These categories were standardized across the research teams. Finally, during the compilation stage, each team conducted a final analysis, summarizing the information extracted for each code in a table. This table was further summarised in a second round to reduce the amount of information. Subsequently, these summaries were combined into a summary encompassing the information from all three countries.

Patient and Public Involvement

Adolescents and young adults (aged 15-24 years old) with knowledge and lived experience of mental distress, including depression and anxiety, were included as part of the Lived Experience Advisory Panel (LEAP) in the three cities where the study was conducted (Buenos Aires, Bogotá, Lima). We received input from the LEAP committee during the design and conduct of the study. The main roles of the LEAP were to provide expert advice to the local research team on all appropriate aspects of the project, including research materials, methods, results, and dissemination.

Ethical statement

The study protocol and all procedures were approved by the Institutional Review Board (IRB) of Universidad de Buenos Aires on February 10th 2020, Pontificia Universidad Javeriana on March 3rd 2020, and Universidad Peruana Cayetano Heredia on March 31st 2020. Furthermore, it was approved by the IRB of Queen Mary University of London on June 5th 2020.

Results

Participants' characteristics

A total of 185 participants took part in this study: 111 participants (36 adolescents, 35 young adults and 40 professionals) attended the 18 focus groups, on average approximately six participants per group in each country; and 74 young people (29 adolescents and 45 young adults) took part in the 12 arts workshops. The average number of participants in the workshops varied across the three countries with five in Argentina, six in Colombia and nine in Peru.

The socio-demographic characteristics of all groups are shown for each city in Tables 1 and 2.

DRAFT For peer review only

For peer review only - http://bmjopen.bmj.com/site/about/guidelines.xhtml

Table 1. Socio-demographic characteristics of young people in focus groups and arts workshops

**Focus Groups**				
	**Buenos Aires**	**Bogotá**	**Lima**	**Total**
**Age group**				
Adolescents	13 (48%)	9 (50%)	14 (54%)	36 (51%)
Young adults	14 (52%)	9 (50%)	12 (46%)	35 (49%)
**Education level completed**				
Primary school	17 (63%)	9 (50%)	14 (54%)	40 (56%)
Secondary school	4 (15%)	6 (33%)	9 (35%)	19 (27%)
Higher/technical education	6 (22%)	-	3 (12%)	9 (13%)
Other	-	3 (17%)	-	3 (4%)
**Previous experience of anxiety and/or depression**				
Yes	17 (63%)	14 (78%)	18 (69%)	49 (69%)
No	10 (37%)	4 (22%)	8 (31%)	22 (31%)
**Previous experiences with mental health treatment of those who reported previous experience of anxiety and/or depression**				
Yes	9 (53%)	11 (79%)	7 (39%)	27 (55%)
No	8 (47%)	3 (21%)	11 (61%)	22 (45%)
**Arts workshops**				
**Age group**				
Adolescents	9 (45%)	11 (30%)	9 (53%)	29 (39%)
Young adults	11 (55%)	26 (70%)	8 (47%)	45 (61%)

Table 2. Socio-demographic characteristics of professionals participating in focus groups

	**Buenos Aires**	**Bogotá**	**Lima**	**Total**

DRAFT

For peer review only

For peer review only - http://bmjopen.bmj.com/site/about/guidelines.xhtml

Psychologists	4 (29%)	4 (29%)	3 (25%)	11 (28%)
Teachers	3 (21%)	6 (43%)	1 (8%)	10 (25%)
Youth workers	3 (21%)	2 (14%)	3 (25%)	8 (20%)
Psychiatrists	2 (14%)	-	-	2 (5%)
Other (e.g., volunteer, researcher, consultant)	2 (14%)	2 (14%)	5 (42%)	7 (23%)

Resources identified

The resources and activities that help young people to recover from mental distress were grouped into 8 categories: I- Personal resources, II- Personal development, III- Spirituality and religion, IV- Social resources, V- Social media, VI- Community resources, VII- Activities (subcategorised into artistic, leisure, sports and outdoor activities), and VIII- Mental health professionals. Due to the large amount of data collected, only the most commonly reported resources are presented here.

I - Personal resources

Young people, adolescents and professionals refer to personal resources as tools to overcome mental health problems. These resources can be allocated to four sub-categories: relationship with oneself, personal traits, coping strategies and passive strategies.

The first sub-category, relationship with oneself, emphasised the importance of self-awareness and self-esteem. Some participants mentioned that having clear goals builds their confidence and helps them overcome problems.

> *"It is very important to value yourself and accept the problem and know how to handle the situation (...) It is also very important to know yourself and know what you like and do not like to know what can help you with the distress" (Female adolescent, Buenos Aires)*

The second subcategory, personal traits, refers to what young people consider to be the key in overcoming mental distress. Most commonly, empathy was mentioned as a helpful trait, but also resilience and motivation.

The third subcategory were coping strategies, including reflecting about problems, accepting and thinking about a difficult situation, controlling emotions and talking to oneself.

The last subcategory 'passive strategies' involved avoiding or isolating themselves from other people when feeling distressed.

DRAFT

For peer review only

For peer review only - http://bmjopen.bmj.com/site/about/guidelines.xhtml

> *"When I feel depressed or sad, I make an abrupt change to the situation, I stop what I am doing, I leave and, for instance, listen to music, draw, or, since generally I do not go out, only sometimes, I prefer to be alone, it is how I feel more peaceful" (Male young adult, Bogotá)*

II- Personal development

Personal development comprises activities related to academic, occupational and/or vocational development. Young adults mentioned work, job placement and entrepreneurship as a helpful resource to cope with difficult situations and stay active and motivated. School attendance was perceived by professionals as a protective factor for adolescents.

> *"Young people without access to school, or outside educational activities, without access to a recreational space, are negatively affected. Economic factors influence a lot and a lack of resources is closely related to the economic situation" (Psychologist, Buenos Aires)*

III- Spirituality and religion

Religion and spirituality were reported as important resources by participants from Bogotá and Lima. For spirituality, some participants from Bogotá stated that meditation as a practice helped them to relax, calm their mind and get rid of negative energy. Some participants from Lima, mostly young women, mentioned exercises related to relaxation, such as breathing exercises, yoga, meditation, and mindfulness.

Religion was suggested as a resource by participants from both cities. In Bogotá, religion was seen as a resource in moments of sadness and depression and a way to seek guidance for solving problems. In Lima, some participants talked about praying or reading the bible and talking to God was regarded in a similar manner as meditating.

> *"I am Christian, but it's not like I go to confession with a priest (laughs). It's more like simply talking with God, I tell Him 'Father, this happened to me'. It's curious because I did not understand very well from a scientific point of view why that happened, but there is research that says if you are talking with someone, even if they don't exist, it is like a direct meditation. That's how I see it." (female, adolescent, Lima)*

Some participants from Bogotá pointed out that religion can make people feel judged or not good enough. Some participants from Bogotá and Lima remarked that young people are not as religious as older people.

IV- Social resources

Social resources focused on receiving support from friends, family and other people, as well as participating in group activities. Participants from all three cities agreed that support from friends

For peer review only - http://bmjopen.bmj.com/site/about/guidelines.xhtml

DRAFT

For peer review only

is an important source of emotional support for young people. Talking about their problems made them feel better and engaging in activities with friends was a welcome distraction. Professionals highlighted friends and social groups as an important resource, in some cases more important than the family.

> *"I believe it is fundamental, the support, the emotional support that family or friends can provide. Like someone mentioned, a lot of the time you confide in your friends more than in your family" (Male young adult, Bogotá)*

Repeatedly, young people referred to the family as an important source of support, providing helpful advice and making them feel calmer and more relaxed. Family support was seen as a valuable resource to navigate situations of emotional distress. Other social sources of support were partners and teachers.

> *"In my case, I talk more with my family, my mom, my sisters, my dad, mostly about things that happen at work, I always talk to them. First, because they are closer to me and because I believe they have the experience to give me advice, with other issues, maybe outside of work, I may talk to friends, but almost always I talk to my family" (Male young adult, Lima)*

Another helpful resource was the sense of belonging to a group with similar interests and with whom they can share experiences and views. Most frequently reported were sports, artistic and religious groups, but political groups and scouts also came up as helpful resources. These groups helped to forget about problems, openly express feelings, feel understood and supported by peers, and develop skills (i.e., artistic skills). Socialising and communicating in these groups could improve the mood in situations of distress.

V- Social media

Social media were reported as a valuable resource to distract themselves, maintain contact with friends and acquaintances, meet new people or watch inspiring content. However, some participants also referred to negative aspects of social media, such as promoting unrealistic expectations about living standards and physical appearance as well increase the risk of being cyberbullied.

> *"It's a bit 50/50, because it depends on the type of people you have on your social network. Why I say this, because in my case, during school, for a long time I was cyber bullied. I had to close my accounts, report it, everything, all bad. And in the same place I found people who supported me and helped me, motivated me and took me through the path of God. I guess it depends on the type of people you have on your social network and how you use it" (Female young adult, Bogotá)*

For peer review only - http://bmjopen.bmj.com/site/about/guidelines.xhtml

VI- Community resources

Community resources were suggested only by participants from Bogotá and Buenos Aires. They included parks, cultural centres, botanical gardens, public libraries and activities such as workshops focused on arts, writing, reading, poetry and sports. Young adults and adolescents highlighted the importance of having access to these spaces to meet other people or engage in activities, which help them forget about their problems.

Professionals stated that these resources and spaces helped young people to develop their identity and build bonds, emphasising more often clubs, schools, and community spaces.

> *"When I go to the cultural centre and dance or when I go to the Scouts on Saturday, I focus so much on that [activity] that I forget about my problems and have a great time" (Female adolescent, Buenos Aires)*

VII- Activities

Arts activities

Arts activities such as playing music, painting, drawing and dance were commonly reported as a resource that allowed participants to meet people with similar interests and express themselves and their emotions freely in a contained context, particularly in situations of discomfort. Such activities would also help to develop social and problem-solving skills, and gain self-knowledge and confidence. Arts activities were perceived liberating and stress reducing. In addition, professionals stated arts enable young people to explore and develop their talents.

In addition to the above mentioned arts activities, writing was suggested as a resource, mostly by women. Some write stories or poems as it allows them to process and express their emotions, while others prefer free writing about their problems or any other topic as a way to relax and see the problems from another point of view. Other less common artistic activities mentioned were theatre, clown, circus, graffiti, rap, sculpture and photography.

> *"It's not just one thing, not just drawing, not just listening to music, not just writing, it's everything that has to do with art, I feel it liberates me a lot, it gives me peace, calms me down when facing situations so complicated, we have. It is like your own psychologist, to whom you can tell how you feel without thinking of others and that is why I like it so much" (Male young adult, Bogotá)*

Leisure activities

Hobbies and enjoyable leisure activities such as listening to music or attending a concert were reported as helping to manage stress, relax, forget about problems and create supportive bonds with others.

For peer review only - http://bmjopen.bmj.com/site/about/guidelines.xhtml

"Whenever I felt sad, I listened [to music], I put on my headphones, put some music on that I know I listened to when I felt happy" (Male young adult, Lima)

Other leisure activities that worked as a welcome distraction from problems and distress were watching television, movies, series, music videos, and broadcasts of sporting events; manual activities such as cooking, knitting, sewing and recycling; playing with pets; reading; listening to podcasts; attending conferences; solving brain teasers; playing games; and going on trips.

Sports activities

A further resource were sports and physical activities which helped to relax, distract, overcome emotional distress and bond with peers.

"When I do sports, the problems leave, you focus only on what you have to do or what you want to do, to improve and be okay. It clears your head a lot and that helps you overcome any problem" (Male adolescent, Buenos Aires)

Outdoor activities

Some participants mentioned outdoor activities as a resource which helped to relax, particularly at times of sadness and distress. These activities included going out for a walk, either alone or in a group, skateboarding and hanging out or sunbathing with friends in the park.

"When I have been in those moments [of mental distress] I went to the park and seat there, close my eyes and just listen to the sounds, the wind, how the plants move" (Female young adult, Lima)

Other outdoor activities were related to contact with nature such as nature walks, going to parks, camping, going to the beach or the riverside.

"I noticed watching nature helped me a lot, it relaxes me and helps me process things. To see the sky and more natural environments, I noticed it helps me greatly." (Female young adult, Bogotá)

VIII- Mental health professionals

As shown in Table 1, more than a third of the young people in the focus groups reported previous experiences with mental health treatment. Their experiences were mostly with psychologists at school, university or youth groups. In Bogotá, some participants also mentioned having participated in support groups and coaching led by psychologists.

The experiences were reported as mostly positive and helpful to solve problems, vent emotions and receive support. Those participants who had never been in contact with a mental health professional, they stated that they would seek contact with a professional only if they had a serious problem, which they could handle on their own.

DRAFT

For peer review only

For peer review only - http://bmjopen.bmj.com/site/about/guidelines.xhtml

"If the problem has gotten out of my hands, I would go to a psychologist, for guidance" (Male young adult, Lima)

Discussion

Main findings

Adolescents and young adults living in large cities in Latin America report a range of resources that can help them to recover from mental distress. Across the capitals in three Latin American countries, similar resources were identified. They were grouped into eight categories: personal resources, personal development, spirituality and religion, social resources, community resources, social media, activities (artistic, leisure, sports and outdoor activities), and mental health professionals. Several of the categories overlap, but all of them focus on different types of resources. In their combination they show a rich picture of potential resources that young people from economically disadvantaged districts in large cities in Latin America use and find helpful to cope with mental distress.

The reported resources address personal aspects such as personal development, the expression of emotions, feeling distracted and the acquisition of skills. Most of the resources however have a strong social element and refer to support received from family, friends and other groups. Various of the most commonly reported activities combine the two aspects: they distract and cheer up the young person, and at the same time they involve being in groups and feeling understood and supported by others.

Strengths and limitations

To our knowledge, this is the first study to explore resources of young people to overcome mental distress in large urban environments across three Latin American countries. Using the same language facilitated the analysis across countries and allowed for direct comparisons. The study captured not only perspectives from three different countries, but also from three groups, i.e. adolescents, young adults and professionals. Moreover, the material was generated through two different methods. We used focus groups as a more conventional form of qualitative approach and structured group conversations embedded in arts workshops which is a more innovative method in this context and provides a different approach to engage young people and encourage them to express their views. Combining the different perspectives and methods yielded comprehensive material with many commonalities, suggesting a validity of the findings.

However, this study also had some limitations. Firstly, and most importantly, focus groups and structured group conversations were conducted online. This was inevitable because of the restrictions due to the pandemic. Although this mode of communication posed fewer practical

For peer review only - http://bmjopen.bmj.com/site/about/guidelines.xhtml

problems than originally expected by the research team and still generated rich material, the online communication may have influenced the interaction between the facilitators and the participants and thus the results. Secondly, the recruitment of a substantial proportion of the participants through arts organisations may have biased the findings, leading to a particularly strong emphasis on arts activities and related group experiences. Thirdly, we conducted the study with young people from economically disadvantaged districts in three large cities in Latin America. The three cities have some commonalities and differences. The cultural, economic and social context in large cities in other Latin American countries may differ so that not all of the findings of this study may be generalisable to cities in those other countries. Fourthly, the study was conducted during the pandemic which may have influenced the views of young people as to what they would find helpful, perhaps emphasising group activities that were difficult to access during that period. Finally, we assessed the views of young people and professionals about helpful resources which may not be representative of the experience of all young people in Latin America and have no evidence as to what extent those resources are actually used and are beneficial in reality.

Comparison with existing literature

Several of the findings can be related to other research and appear largely consistent with what has been found in other studies before. Activities related to academic, occupational or vocational development such as work, job placement and entrepreneurship were considered as a helpful resource to cope with difficult situations and to help young people stay active and motivated. These findings may be related to the lower rates of anxiety and depressive disorders found in adolescents and young adults with higher educational levels in a study in Chile (30).

Receiving support from friends and belonging to a group were considered an important social resource (20). Whilst family was also reported as a helpful resource, this was not as consistently mentioned as one might have expected given the cultural importance of the family in the Latin American context (31). One can only speculate as to whether this was because of the age of the participants who – as adolescents and young adults – were in a phase of life where they take a distance from their family of origin and have not yet founded a new family themselves (32).

Arts activities and sports were two resources frequently reported as helpful to improve mental health. Activities such as painting, dance, music, handcrafts and other creative arts were identified as a form of non-verbal expression of feelings that improves confidence and helps to reduce distress. Several studies have identified the benefits of these types of activities for young people's mental health. A systematic review showed that artistic activities are beneficial as they allow participants to communicate through art, express thoughts and feelings more effectively, increase self-esteem and improve the ability to handle anger (33). Another study showed that teens who take part in weekly extracurricular activities, including art, had less anxiety and depression and were generally more optimistic (34). Similarly, participants suggested that physical activities such

For peer review only - http://bmjopen.bmj.com/site/about/guidelines.xhtml

as running, and team sports were beneficial to reduce stress and improve energy and mood. This finding is consistent with the results of a systematic review showing sports are associated with an improvement in psychosocial health (35).

Implications

The findings may have several implications. For reducing anxiety and depression of an individual or on a group level, not all of the resources listed in this study are likely to be equally available or relevant in a given context However, the eight categories may provide a framework that can help to consider different options for developing programmes that facilitate and strengthen some of the individual and social resources and activities that may help young people to overcome their distress in lower income urban settings.

The findings show that young people come up with a wide range of ideas and experiences about helpful resources. Thus, when considering approaches to improve the mental health of young people, their voice should be heard and take a central role in the development of policies (36). When their views are elicited, innovative methods like arts workshops may help to engage young people and generate helpful material. Based on the experiences in this study, conducting such workshops and interviews online appears a feasible and valuable method to provide a range of findings. Online methods may help to complete similar studies with fewer resources and in shorter periods of time than studies with conventional in-person interviews, thus making them more feasible, particularly in low-resource settings.

Most of the young people in our study reported to have experienced anxiety and/or depression and more than a third had received some type of treatment, although there is no information about the type of treatment. In any case, young people reported largely positive experiences of mental health professionals. Future policies to improve the mental health of young people in large cities should address the need for accessible and effective mental health services. However, this cannot be the sole focus as the provision of mental health services requires substantial funding and using professionals can come with the implicit message that the affected young person cannot cope on their own, which in turn may undermine confidence rather than strengthen it. On a population level, promoting personal and interpersonal skills at school, support from peers and groups, and access to various activities appear more important to reduce the overall level of mental distress in young people.

Finally, considering that this study was carried out in urban environments, it is important to stress the value of community spaces where young people are able to play sports, meet, engage in enjoyable group activities and distract themselves such as parks, public libraries or theatres. In addition to the accessibility of appropriate space, there should also be community organisations enabling and facilitating different activities, especially since some sports and arts activities require

For peer review only - http://bmjopen.bmj.com/site/about/guidelines.xhtml
DRAFT
For peer review only

equipment and guidance. Future research may explore to what extent community organisations should be set up and supported to attract, engage and help particularly vulnerable and mentally distressed young people.

Conclusion

Anxiety and depression are frequent among young people. This study shows that in a challenging context like large cities in Latin America, there is a wide range of resources, similar across the three cities, that young people can use and report as helpful. Future research and policies may focus on how these resources can be made widely available, be strengthened and be flexibly utilised by young people if and when required.

Declarations:

Ethics approval and consent to participate:

Written approval was received for this study from the Faculty of Medicine - Research and Ethics Committee of the Pontificia Universidad Javeriana, Bogota on 20/03/2020 (FM-CIE-0241-20) from the Institutional Ethics Committee on Research of the Universidad Peruana Cayetano Heredia, Lima on 31/03/2020 (Constancia E021-03-20), and from the Queen Mary Ethics of Research Committee on 16/11/2020 (QMERC2020/02). All methods were carried out in accordance with relevant guidelines and regulations. All informed consent/assent was obtained from all subjects and/or their legal guardian(s) for participants aged less than 16 years.

Availability of data and materials:

The consent forms did not specify that the data would be deposited in a public repository, and for this reason we are unable to deposit the data. The de-identified participant dataset will be made available from the corresponding author (Natalia Godoy Casasbuenas, natalia.godoy@javeriana.edu.co) upon reasonable request and subject to a data sharing agreement.

Funding:

This work is supported by the Medical Research Council (grant number: MR/S03580X/1)

Authors' contributions:

SP, LIB, FDC, CGR, JMUR and PH devised the project, the main conceptual ideas and design of the study.

MT, NGC, NO, FC, LHP, CGR, JMUR, MS and FDC participated in data collection during the artistic workshops and focus groups.

MT, NGC, LHP, NO, FC participated in the transcription and data analysis of the structured group conversations and focus groups.

MT, NGC, NO wrote the manuscript with input from all authors.

Competing interests: None declared

For peer review only - http://bmjopen.bmj.com/site/about/guidelines.xhtml

Acknowledgements:

We thank Santiago Lucchetti, Jorge Pasquini, Natalia Ortiz, Karen Ariza and Diliniya Stanislaus Sureshkumar for their contributions in the analysis stage. We thank Victoria Jane Bird for comments on the manuscript.

We would like to acknowledge our arts organisations for their contribution to the OLA programme, Crear Vale La Pena, Fundación Batuta, Familia Ayara, Arenas y Esteras and Teatro La Plaza.

References

1. Somerville LH, Jones RM, Casey BJ. A time of change: behavioral and neural correlates of adolescent sensitivity to appetitive and aversive environmental cues. Brain Cogn. 2010 Feb;72(1):124–33.

2. Spear LP. The adolescent brain and age-related behavioral manifestations. Neurosci Biobehav Rev. 2000 Jun;24(4):417–63.

3. Lopez V, Sanchez K, Killian MO, Eghaneyan BH. Depression screening and education: an examination of mental health literacy and stigma in a sample of Hispanic women [Internet]. Vol. 18, BMC Public Health. 2018. Available from: http://dx.doi.org/10.1186/s12889-018-5516-4

4. van der Heide I, Wang J, Droomers M, Spreeuwenberg P, Rademakers J, Uiters E. The relationship between health, education, and health literacy: results from the Dutch Adult Literacy and Life Skills Survey. J Health Commun. 2013;18 Suppl 1:172–84.

5. Benjet C, Sampson L, Yu S, Kessler RC, Zaslavsky A, Evans-Lacko S, et al. Associations between neighborhood-level violence and individual mental disorders: Results from the World Mental Health surveys in five Latin American cities. Psychiatry Res. 2019 Dec;282:112607.

6. Morgan C, Reininghaus U, Reichenberg A, Frissa S, SELCoH study team, Hotopf M, et al. Adversity, cannabis use and psychotic experiences: evidence of cumulative and synergistic effects. Br J Psychiatry. 2014 Mar 13;204:346–53.

7. World Health Organization. Adolescent mental health. Fact sheet [internet]. 2021 [updated 2021 November 17; cited 2021 November 30]. Available from: https://www.who.int/news-room/fact-sheets/detail/adolescent-mental-health

8. Adwas A, Jbireal J, Azab A. Anxiety: Insights into Signs, Symptoms, Etiology, Pathophysiology, and Treatment. The South African journal of medical sciences. 2019;2:80-91.

For peer review only - http://bmjopen.bmj.com/site/about/guidelines.xhtml

9. Caqueo-Urízar A, Flores J, Escobar C, Urzúa A, Irarrázaval M. Psychiatric disorders in children and adolescents in a middle-income Latin American country [Internet]. Available from: http://dx.doi.org/10.21203/rs.2.14739/v2

10. Van Ameringen M, Mancini C, Farvolden P. The impact of anxiety disorders on educational achievement. Journal of anxiety disorders. 2003 Jan 1;17(5):561-71.

11. Patel V, Flisher AJ, Hetrick S, McGorry P. Mental health of young people: a global public-health challenge. The Lancet. 2007 Apr 14;369(9569):1302-13.

12. Kim D. Blues from the neighborhood? Neighborhood characteristics and depression. Epidemiologic reviews. 2008 Nov 1;30(1):101-17.

13. Morgan C, Reininghaus U, Reichenberg A, Frissa S, Hotopf M, Hatch SL, SELCoH Study Team. Adversity, cannabis use and psychotic experiences: evidence of cumulative and synergistic effects. The British Journal of Psychiatry. 2014 May;204(5):346-53.

14. Thapar A, Collishaw S, Pine DS, Thapar AK. Depression in adolescence. The Lancet. 2012 Mar 17;379(9820):1056-67.

15. Murcia EH, Losada YL, Rojas LA, Londoño PG. Prevalencia de la depresión infantil en Neiva (Colombia). Avances en Psicología Latinoamericana. 2009 Sep 23;27(1):154-64.

16. Méndez X, Rosa AJ, Montoya M, Espada JP, Olivares J, Sánchez-Meca J. Tratamiento Psicológico de la Depresión Infantil. Psicología conductual. 2002;10(3):563-80.

17. March J, Silva S, Petrycki S, Curry J, Wells K, Fairbank J, et al. Fluoxetine, cognitive-behavioral therapy, and their combination for adolescents with depression: Treatment for Adolescents With Depression Study (TADS) randomized controlled trial. JAMA [Internet]. 2004 Aug 18 [cited 2021 Jul 12];292(7). Available from: https://pubmed.ncbi.nlm.nih.gov/15315995/

18. Stapinski LA, Montgomery AA, Heron J, Jerrim J, Vignoles A, Araya R. Depression symptom trajectories and associated risk factors among adolescents in Chile. PLoS One. 2013 Oct 11;8(10):e78323.

19. Anderson K, Priebe S. Concepts of resilience in adolescent mental health research. Journal of Adolescent Health. 2021 May 24.

20. Ayed N, Toner S, Priebe S. Conceptualizing resilience in adult mental health literature: A systematic review and narrative synthesis. Psychol Psychother. 2019 Sep;92(3):299–341.

21. Zarobe L, Bungay H. The role of arts activities in developing resilience and mental wellbeing in children and young people a rapid review of the literature [Internet]. Vol. 137, Perspectives in Public Health. 2017. p. 337–47. Available from: http://dx.doi.org/10.1177/1757913917712283

22. Hildebrand NA, Celeri EHRV, Morcillo AM, Zanolli M de L. Resilience and mental health problems in children and adolescents who have been victims of violence. Rev Saude Publica.

For peer review only - http://bmjopen.bmj.com/site/about/guidelines.xhtml

2019 Jan 31;53:17.

23. Suarez EB. Two decades later: The resilience and post-traumatic responses of Indigenous Quechua girls and adolescents in the aftermath of the Peruvian armed conflict. Child Abuse Negl. 2013 Feb;37(2-3):200–10.

24. Vera-Bachmann D. [Resilience, Poverty and Rurality]. Rev Med Chil. 2015 May;143(5):677–8.

25. Herrera-Moreno D, Carvajal-Ovalle D, Cueva-Nuñez MA, Acevedo C, Riveros-Munévar F, Camacho K, et al. Body image, perceived stress, and resilience in military amputees of the internal armed conflict in Colombia. Int J Psychol Res (Medellin). 2018 Jul;11(2):56–62.

26. Miller-Graff LE, Scheid CR, Guzmán DB, Grein K. Caregiver and family factors promoting child resilience in at-risk families living in Lima, Peru. Child Abuse Negl. 2020 Oct;108:104639.

27. Creswell JW, Hanson WE, Clark Plano VL, Morales A. Qualitative research designs: Selection and implementation. The counseling psychologist. 2007; 35(2):236-264.

28. Priebe S, Fung C, Brusco LI, Carbonetti F, Gómez-Restrepo C, Uribe M, Diez-Canseco F, Smuk M, Holt N, Kirkbride JB, Araya R. Which resources help young people to prevent and overcome mental distress in deprived urban areas in Latin America? A protocol for a prospective cohort study. BMJ open. 2021 Sep 1;11(9):e052339.

29. Bengtsson M. How to plan and perform a qualitative study using content analysis. NursingPlus Open. 2016 Jan 1;2:8-14.

30. Gaete J, Labbé N, Del Villar P, Allende C, Araya R, Valenzuela E. Mental health and associated factors among young offenders in Chile: a cross-sectional study. Crim Behav Ment Health. 2018 Feb;28(1):13–27.

31. Kuznesof EA. The House, the Street, Global Society: Latin American Families and Childhood in the Twenty-First Century [Internet]. Vol. 38, Journal of Social History. 2005. p. 859–72. Available from: http://dx.doi.org/10.1353/jsh.2005.0065

32. Parra A, Oliva A, del Carmen Reina M. Family Relationships From Adolescence to Emerging Adulthood [Internet]. Vol. 36, Journal of Family Issues. 2015. p. 2002–20. Available from: http://dx.doi.org/10.1177/0192513x13507570

33. Uttley L, Scope A, Stevenson M, Rawdin A, Taylor Buck E, Sutton A, et al. Systematic review and economic modelling of the clinical effectiveness and cost-effectiveness of art therapy among people with non-psychotic mental health disorders. Health Technol Assess. 2015 Mar;19(18):1–120, v – vi.

34. Thomson LJ, Morse N, Elsden E, Chatterjee HJ. Art, nature and mental health: assessing the biopsychosocial effects of a “creative green prescription” museum programme involving horticulture, artmaking and collections. Perspect Public Health. 2020 Sep;140(5):277–85.

DRAFT
For peer review only

35. Eime RM, Young JA, Harvey JT, Charity MJ, Payne WR. A systematic review of the psychological and social benefits of participation in sport for children and adolescents: informing development of a conceptual model of health through sport. Int J Behav Nutr Phys Act. 2013 Aug 15;10:98.

36. Oxman AD, Lewin S, Lavis JN, Fretheim A. SUPPORT Tools for evidence-informed health Policymaking (STP) 15: Engaging the public in evidence-informed policymaking. Health Res Policy Syst. 2009 Dec 16;7 Suppl 1:S15.

DRAFT
For peer review only

For peer review only - http://bmjopen.bmj.com/site/about/guidelines.xhtml

OLA WP1 Arts-based workshops: Topic guide for structured conversations with adolescents and young adults

Objectives: (1) To explore the experiences of emotional and mental health problems, including feelings of depression, anxiety, and stress of young people with emphasis on understanding how these episodes have been recovered and overcome. (2) Explore what role they think arts and creativity had or could have had to protect them and help them recover from anxiety and depression.

a. Breaking the ice

What caught your attention in the theatre?
If theatre is removed from your life, what would you lose?

b. Recognizing mental anguish

We want to know what emotional and mental health problems (anxiety / depression / stress) mean for you.

Have you ever experienced it? If not, have you perceived it in people close to you?

How do you experience it? Can you visualize / express it? Can you communicate it to others? Have you found metaphors or symbols or arts to express these feelings?

Have you found any artistic resource to express these feelings, such as workshops, poetry, music, etc?

c. Coping with mental anguish

Where do you go when you feel distressed? Real places? Fantasy worlds? Are you looking for help in your environment? Do you attend reflection groups? Individual therapy? Group therapy? Do you go to Church? Do you play sport or take physical exercise? Or do you take refuge in yourselves? Do you use your imagination? Do you make art?

How do you physically experience anxiety / distress? What other impact does it have on your life?

Who do they talk to about these feelings? Other young people? Friends? Teachers? Families? Where? When? Who can help best in these moments?

Can you map out the resources (people / places / activities) in your daily life that allow you to reduce distressed state and acquire greater strength in triggering situations? What keeps you safe and comfortable? What significant physical and mental changes do you think you have experienced? How could them describe this mood change?

For peer review only

The recovery process is not always linear or progressive, it involves progress and Setbacks. Can you identify what are the situations or elements that influence those setbacks?

What emotions or feelings do you perceive when moving from anxiety to emotional stability?

d. Understanding the relationship between art and resilience

How do the arts and creative practices build your ability to recover from anxiety and distress as well as to develop resilience to mental pathology?

For peer review only

OLA WP1: Topic guide for focus groups with adolescents, young adults and professionals to explore views on resources that young people use to overcome mental distress

Introduction and group rules (5 mins)

Thank participants for their availability, introduce researchers and explain:

- All participants here have signed a consent form that describes the study and the aim of the group.
- Aim of the group: to explore and understand your views on the types of resources that young people use to overcome mental and emotional health problems. By resources we mean what helps people to recover, specifically personal resources (for example, what people do and how people might cope in different situations), and social resources (for example, your friendships).
- We are interested in seeing if the resources that young people use to recover from mental and emotional health problems change as they grow older; and so we are involving young people in our study who are aged 15-16 and 20-24 years.
- Confidentiality: the names of the participants will only be known by the researchers and other participants in today's focus group, and will not be revealed to anyone else.
- The session will be audio-recorded, transcribed and analysed by researchers.
- All participants will be assigned an ID number and all potentially identifying information will be removed.
- Audio recordings will be destroyed once data analysis is complete.
- One researcher will facilitate the discussion using a topic guide. The second researcher will make sure all areas to be discussed are covered and to write notes about important points raised.

<u>Outline group rules</u>:

- Information shared within the group must remain confidential. We ask that you try not to use names if talking about services or staff members.
- Speaking one at a time and listening and respecting others' opinions
- You do not have to share information that you do not want to
- There are no right or wrong answers
- We are looking for the widest possible range of views. Alignment of views within the group is not required
- Mobile phones to be switched off or on silent mode (unless required for work/urgent personal reasons)

- Any questions?
- Start audio recorder

Ice breaker (approx. 5 mins):

Ask each participant in turn to say:

For peer review only

- Their name or nickname
- And something about themselves, e.g. one thing they enjoy doing or a talent they have

Focus group discussion (approx. 45 mins):
Aim: to explore and understand the views and experiences of adolescents, young adults and professionals on the types of resources that young people use, alternative ways of measuring resources and on the resources included in the assessment battery.

During adolescence and youth, it can be common for people to experience mental and emotional health problems, which includes feelings of depression, anxiety and stress. Living in big cities can mean people are more likely to experience stressful events. However, not all people develop mental and emotional health problems, and when people do become emotionally distressed, many will recover within a year. And so we want to understand what helps people to recover.

<ins>Introduction:</ins>

- <ins>What are the situations or experiences that might cause emotional distress in young people?</ins>

<ins>Types of resources</ins>:

- What helps young people to recover from mental and emotional health problems?
- What resources do young people have in their daily life that help with feelings of emotional distress?
- What helps people if they are feeling down, upset or anxious?

Prompts:
Family/partner
Friendships
Groups in community such as religious groups, youth centres
Sports activities – alone or in groups
Arts activities – alone or in groups
Internet/social media
Personal resources (thinking styles, coping strategies, self-care)
Green spaces in cities/going out of the city/contact with nature
Can you think of other things that help people?

- How do they help?
  Prompts:
  Do they help all people, or just some people?
  - Are there resources that are more helpful for young people depending on their gender?
  - What resources are more helpful for young people of male/female/non binary gender?
  - Are there resources that are more helpful for your age group compared to the younger/older age group?

- Are there resources in the local community for young people?

For peer review only

- What type of resources

- How do young people learn about and access resources in the local community?
  - How do people find out about these resources?
  - Do they need a referral to start attending a group, club or organisation?
  - Do they need to pay a fee?

- What are your/young people's experiences of accessing these resources?
  Prompts:
  Was it easy to access them and why?
  Did you face any barriers or difficulties?
- What resources would young people like to see and access in the local community?

<u>Measuring resources</u>:
In this research study, we would like to understand better the resources that help young people to overcome mental and emotional health problems. To help with this, we have put together a booklet to ask young people about the resources that they use. The booklet consists of a range of questionnaires that can be completed by young people. At the moment, the resources that are included in the booklet are: (show and read through list of resources).

- In your opinions, are there resources or areas that are important for mental and emotional health to young people, which are missing from the booklet?
  - What resources?
  - Why?
  - How would you ask young people about these resources?

- Are there resources in the booklet, which you think are not relevant for young people?
  - What resources?
  - Why?

- Do you have any other thoughts or comments about the resources in the booklet?

- We currently plan to ask young people about these resources using a booklet of questionnaires. What do you think about this approach?
  - How should young people complete this booklet of questionnaires? (by themselves, with the help of a researcher?)

- What other ways do you think we could use to identify and ask about the resources that young people use, apart from this booklet?
  - Can you think of other approaches to explore resources with young people?

Conclusion (up to 10 mins)

For peer review only

- Does anyone have anything else to add to what has been discussed today: any further comments or recommendations about resources for recovering from mental and emotional health problems?
- Does anyone have questions for us about this study?
- Thank participants for their contribution

For peer review only

Standards for Reporting Qualitative Research (SRQR)*

http://www.equator-network.org/reporting-guidelines/srqr/

Title and abstract

	Page/line no(s).
**Title** - Concise description of the nature and topic of the study Identifying the study as qualitative or indicating the approach (e.g., ethnography, grounded theory) or data collection methods (e.g., interview, focus group) is recommended	Page 0 – Line 1
**Abstract** - Summary of key elements of the study using the abstract format of the intended publication; typically includes background, purpose, methods, results, and conclusions	Page 1

Introduction

**Problem formulation** - Description and significance of the problem/phenomenon studied; review of relevant theory and empirical work; problem statement	Page 3
**Purpose or research question** - Purpose of the study and specific objectives or questions	Page 3 – Lines 30-32

Methods

**Qualitative approach and research paradigm** - Qualitative approach (e.g., ethnography, grounded theory, case study, phenomenology, narrative research) and guiding theory if appropriate; identifying the research paradigm (e.g., postpositivist, constructivist/ interpretivist) is also recommended; rationale**	Page 3 – Line 35
**Researcher characteristics and reflexivity** - Researchers' characteristics that may influence the research, including personal attributes, qualifications/experience, relationship with participants, assumptions, and/or presuppositions; potential or actual interaction between researchers' characteristics and the research questions, approach, methods, results, and/or transferability	Page 5 – Lines 22-24
**Context** - Setting/site and salient contextual factors; rationale**	Page 4 – Lines 26-29
**Sampling strategy** - How and why research participants, documents, or events were selected; criteria for deciding when no further sampling was necessary (e.g., sampling saturation); rationale**	Page 4 – Lines 26-29
**Ethical issues pertaining to human subjects** - Documentation of approval by an appropriate ethics review board and participant consent, or explanation for lack thereof; other confidentiality and data security issues	Page 6 – Lines 17-21
**Data collection methods** - Types of data collected; details of data collection procedures including (as appropriate) start and stop dates of data collection and analysis, iterative process, triangulation of sources/methods, and modification of procedures in response to evolving study findings; rationale**	Page 4 – Lines 11-25<br>Page 5 – Lines 14-34<br>Page 6 – Lines 1-8

For peer review only - http://bmjopen.bmj.com/site/about/guidelines.xhtml

**Data collection instruments and technologies** - Description of instruments (e.g., interview guides, questionnaires) and devices (e.g., audio recorders) used for data collection; if/how the instrument(s) changed over the course of the study	Page 5 – Lines 6-13
**Units of study** - Number and relevant characteristics of participants, documents, or events included in the study; level of participation (could be reported in results)	Page 6 – Lines 23-31
**Data processing** - Methods for processing data prior to and during analysis, including transcription, data entry, data management and security, verification of data integrity, data coding, and anonymization/de-identification of excerpts	Page 5 – Lines 26-27
**Data analysis** - Process by which inferences, themes, etc., were identified and developed, including the researchers involved in data analysis; usually references a specific paradigm or approach; rationale**	Page 5 – Lines 25-34<br>Page 6 – Lines 1-8
**Techniques to enhance trustworthiness** - Techniques to enhance trustworthiness and credibility of data analysis (e.g., member checking, audit trail, triangulation); rationale**	Page 5 – Lines 29-34<br>Page 6 – Lines 1-4

Results/findings

**Synthesis and interpretation** - Main findings (e.g., interpretations, inferences, and themes); might include development of a theory or model, or integration with prior research or theory	Pages 8-12
**Links to empirical data** - Evidence (e.g., quotes, field notes, text excerpts, photographs) to substantiate analytic findings	Pages 8-12

Discussion

**Integration with prior work, implications, transferability, and contribution(s) to the field -** Short summary of main findings; explanation of how findings and conclusions connect to, support, elaborate on, or challenge conclusions of earlier scholarship; discussion of scope of application/generalizability; identification of unique contribution(s) to scholarship in a discipline or field	Pages 12-15
**Limitations** - Trustworthiness and limitations of findings	Pages 13-14

Other

**Conflicts of interest** - Potential sources of influence or perceived influence on study conduct and conclusions; how these were managed	None declared Page 17 Line 37
**Funding** - Sources of funding and other support; role of funders in data collection, interpretation, and reporting	Page 16 – Lines 21-22

*The authors created the SRQR by searching the literature to identify guidelines, reporting standards, and critical appraisal criteria for qualitative research; reviewing the reference lists of retrieved sources; and contacting experts to gain feedback. The SRQR aims to improve the transparency of all aspects of qualitative research by providing clear standards for reporting qualitative research.

For peer review only

**The rationale should briefly discuss the justification for choosing that theory, approach, method, or technique rather than other options available, the assumptions and limitations implicit in those choices, and how those choices influence study conclusions and transferability. As appropriate, the rationale for several items might be discussed together.

Reference:

O'Brien BC, Harris IB, Beckman TJ, Reed DA, Cook DA. **Standards for reporting qualitative research: a synthesis of recommendations.** *Academic Medicine*, Vol. 89, No. 9 / Sept 2014 DOI: 10.1097/ACM.0000000000000388

For peer review only

For peer review only - http://bmjopen.bmj.com/site/about/guidelines.xhtml
